# Nitrogen dioxide decline and rebound observed by GOME-2 and TROPOMI during COVID-19 pandemic

**DOI:** 10.1007/s11869-021-01046-2

**Published:** 2021-08-28

**Authors:** Song Liu, Pieter Valks, Steffen Beirle, Diego G. Loyola

**Affiliations:** 1 Deutsches Zentrum für Luft- und Raumfahrt (DLR), Institut für Methodik der Fernerkundung (IMF), Oberpfaffenhofen, Germany; 2 Present Address: School of Environmental Science and Engineering, Southern University of Science and Technology, Shenzhen, China; 3 Max Planck Institute for Chemistry, Mainz, Germany

**Keywords:** COVID-19, Tropospheric NO_2_, GOME-2, TROPOMI, Harmonized retrieval

## Abstract

Since its first confirmed case in December 2019, coronavirus disease 2019 (COVID-19) has become a worldwide pandemic with more than 90 million confirmed cases by January 2021. Countries around the world have enforced lockdown measures to prevent the spread of the virus, introducing a temporal change of air pollutants such as nitrogen dioxide (NO_2_) that are strongly related to transportation, industry, and energy. In this study, NO_2_ variations over regions with strong responses to COVID-19 are analysed using datasets from the Global Ozone Monitoring Experiment-2 (GOME-2) sensor aboard the EUMETSAT Metop satellites and TROPOspheric Monitoring Instrument (TROPOMI) aboard the EU/ESA Sentinel-5 Precursor satellite. The global GOME-2 and TROPOMI NO_2_ datasets are generated at the German Aerospace Center (DLR) using harmonized retrieval algorithms; potential influences of the long-term trend and seasonal cycle, as well as the short-term meteorological variation, are taken into account statistically. We present the application of the GOME-2 data to analyze the lockdown-related NO_2_ variations for morning conditions. Consistent NO_2_ variations are observed for the GOME-2 measurements and the early afternoon TROPOMI data: regions with strong social responses to COVID-19 in Asia, Europe, North America, and South America show strong NO_2_ reductions of $\sim $30–50% on average due to restriction of social and economic activities, followed by a gradual rebound with lifted restriction measures.

**Supplementary Information:**

The online version contains supplementary material available at 10.1007/s11869-021-01046-2.

## Introduction

On 31 December 2019, an infectious pneumonia of unknown cause, subsequently named as coronavirus disease 2019 (COVID-19), was detected in Wuhan in China. The COVID-19 outbreak was announced as a pandemic in mid-March 2020 and has caused more than 90 million confirmed cases and more than 1.5 million deaths around the world as of January 2021 (https://coronavirus.jhu.edu/map.html). In an effort to prevent the wide and rapid spread of the novel severe virus, countries have imposed national or local restrictions, such as ordering to stay at home, banning on public gathering, and closing non-essential shops and services.

The slowdown and recovery in social and economic activities around the world usually introduce a temporal change of air pollution, particularly for air pollutants strongly related to transportation, industry, and energy. Nitrogen dioxide (NO_2_) is one of the most important and prominent air pollutants affecting human health and ecosystem. Large amounts of NO_2_ are produced anthropogenically in the boundary layer by industrial processes, power generation, transportation, and biomass burning over polluted hotspots. The relatively short atmospheric lifetime of NO_2_ (hours near the surface) facilitates establishing a direct link between observed tropospheric NO_2_ columns and emissions strengths (Richter 2009; Seinfeld and Pandis 2016).

A global and continuous monitoring of atmospheric NO_2_ abundances has been provided by European spaceborne instruments, such as Global Ozone Monitoring Experiment (GOME) aboard ERS-2 (Burrows et al. 1999), Scanning Imaging Absorption SpectroMeter for Atmospheric CHartographY (SCIAMACHY) aboard Envisat (Bovensmann et al. 1999), Ozone Monitoring Instrument (OMI) aboard EOS-Aura (Levelt et al. 2006), Global Ozone Monitoring Experiment-2 (GOME-2) aboard Metop (Callies et al. 2000; Munro et al. 2016), and TROPOspheric Monitoring Instrument (TROPOMI) aboard Sentinel-5 Precursor (Veefkind et al. 2012). The GOME-2 instruments have been providing global pictures of the atmospheric composition since 2007 and will extend this unique dataset until 2027. GOME-2 provides morning observations of NO_2_ at $\sim $09:30 local time, which complement early afternoon measurements from OMI or TROPOMI at $\sim $13:30 local time. The long-term GOME-2 measurements have been widely used in trend studies (Mijling et al. 2013; Hilboll et al. 2013; 2017), satellite dataset intercomparisons (Irie et al. 2012; Krotkov et al. 2017), and emission estimations (Gu et al. 2014; Miyazaki et al. 2017; Ding et al. 2017). The TROPOMI sensor, launched in 2017 with an unprecedented spatial resolution of 7 km×3.5 km (5.5 km×3.5 km after August 2019, van Geffen et al. (2020a)), allows local studies of distribution and evolution of NO_2_ (Stavrakou et al. 2020; Goldberg et al. 2020a; Georgoulias et al. 2020) and regional emission estimates (Lorente et al. 2019; Beirle et al. 2019; van der A et al. 2020; Huber et al. 2020).

**Fig. 1:**
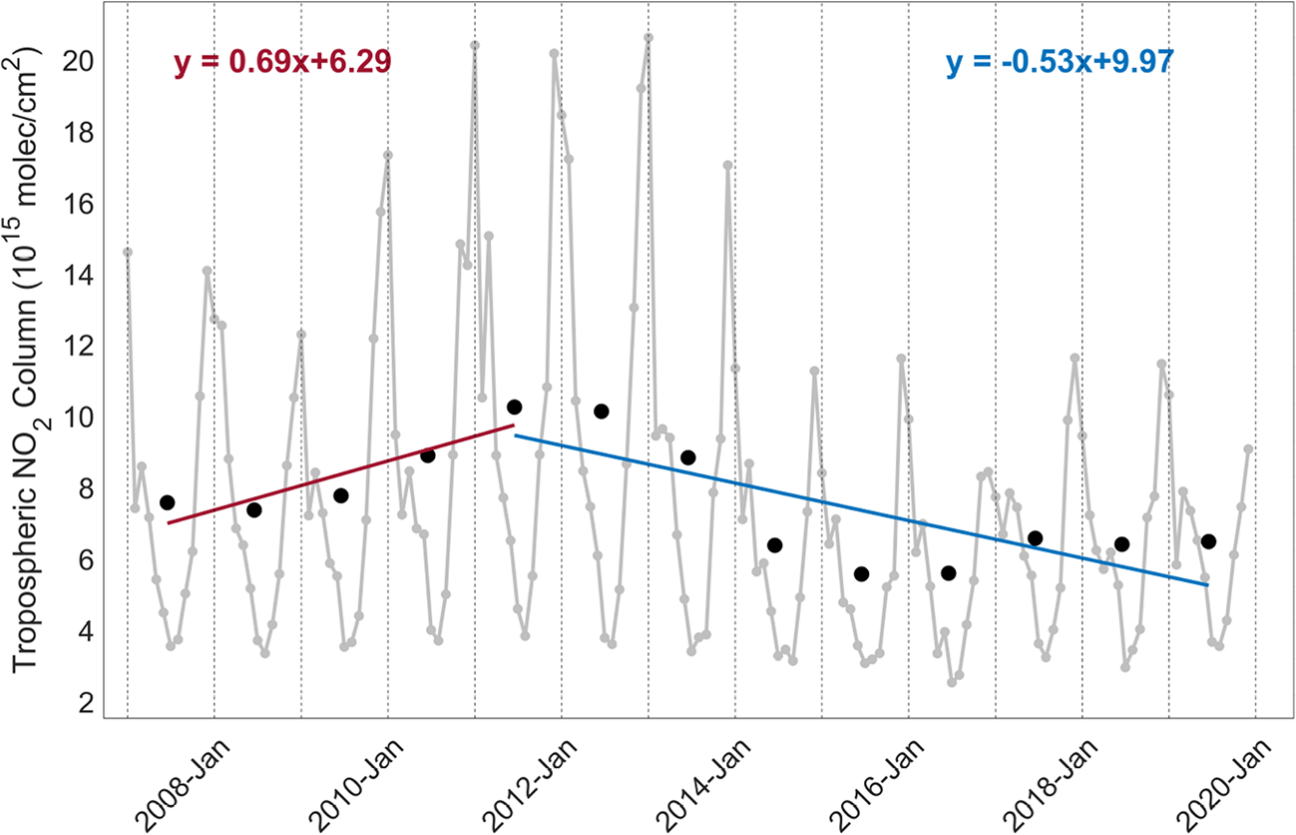
Time series of monthly (gray line) and yearly (black dots) mean tropospheric NO_2_ columns measured by GOME-2A over eastern China (21^∘^N–41^∘^N, 110^∘^E–122^∘^E). The linear fitting result for the yearly average shows a growth for 2007–2011 (red line) and a reduction for 2011–2019 (blue line). Slopes are 0.69 for 2007–2011 (red text) and − 0.53 for 2011–2019 (blue text)

**Fig. 2:**
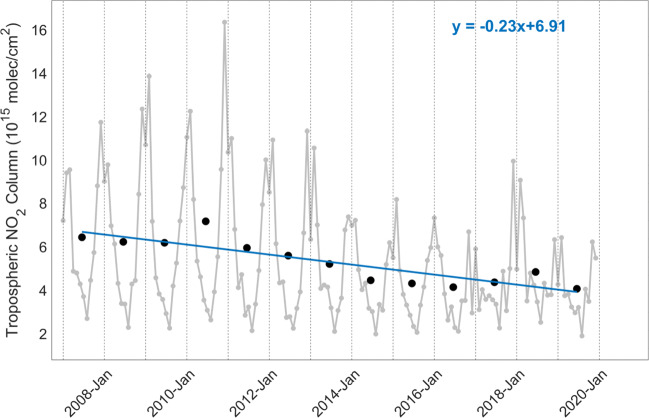
Time series of monthly (gray line) and yearly (black dots) mean tropospheric NO_2_ columns measured by GOME-2A over northern Italy (45^∘^N–46.5^∘^N, 7^∘^E–13^∘^E). The linear fitting result for the yearly average shows a decline (blue line) with a slope of − 0.23

The analysis of NO_2_ concentration variations, however, is not straightforward due to the strong dependency on meteorological conditions, such as solar irradiance and wind fields. Figures 1 and 2 present two examples of NO_2_ monthly variations (gray lines) measured by GOME-2 on MetOp-A for eastern China (21^∘^N–41^∘^N, 110^∘^E–122^∘^E) and northern Italy (45^∘^N–46.5^∘^N, 7^∘^E–13^∘^E) in 2007–2019. The tropospheric NO_2_ columns are generally higher in winter due to the use of combustion power plants for heating and due to the fact that the lower solar irradiances increase the lifetime of NO_2_ in the atmosphere. In addition, the tropospheric NO_2_ columns change over short timescales (hours and days) depending on wind speeds and wind directions that interact with the physical features of the landscape to determine the movement and dispersal of air pollutants. Therefore, NO_2_ changes are typically analyzed using values averaged over long timeframes (months, seasons, and years) based on a long-term satellite dataset (Hilboll et al. 2013; Duncan et al. 2016; Georgoulias et al. 2019). To improve the robustness of the derived temporal changes, chemical transport models (Liu et al. 2020a; Koukouli et al. 2021) or comprehensive statistical models (Hayn et al. 2009; Zhou et al. 2012) can be additionally used.

Based on the spaceborne NO_2_ data from OMI and TROPOMI, recent works have reported the decrease of NO_2_ concentration during the COVID-19 pandemic lockdown across the world (Bauwens et al. 2020). For instance, decreases of tropospheric NO_2_ columns by up to 70% are observed for Chinese populated regions (Fan et al. 2020; Huang and Sun 2020) partly attributed to the decline in anthropogenic emissions related to the COVID-19 crisis (Ding et al. 2020; Zhang et al. 2020). Similar strong decreases by up to 60% are visible in regions with high population and heavy industry, such as India (Singh and Chauhan 2020), southern Europe (Chen et al. 2020; Baldasano 2020), the western USA (Liu et al. 2020b), and South America (Nakada and Urban 2020; Zalakeviciute et al. 2020). These studies generally calculate the weekly or monthly averages of NO_2_ data during the COVID-19 lockdown in 2020 and compare to the same timeframe within recent 5 years or to the period prior to the lockdown in 2020. In addition, the importance of the meteorological variations between years has been explored in regional studies for China (Liu et al. 2020a; Zhao et al. 2020) and the USA (Goldberg et al. 2020b), which can affect the NO_2_ variations by 15%.

In this work, we present an analysis of the NO_2_ variations due to enacting and lifting restrictions on movements in response to the COVID-19 outbreak, covering the severely affected countries across the polluted continents. Long-term NO_2_ measurements from the satellite instrument GOME-2 and high-resolution observations from TROPOMI are applied, with corrections for trend, season, and meteorology. Compared to previous studies, the synergy between morning and early afternoon satellite NO_2_ observations is explored. The GOME-2 and TROPOMI measurements are retrieved in a consistent manner. The time series of morning NO_2_ columns derived from GOME-2 spans over $\sim $14 years of observations.

In “Spaceborne NO_2_ measurements,” the GOME-2 and TROPOMI instruments and the algorithm for tropospheric NO_2_ column retrieval are briefly introduced, followed by a description of the correction method. “COVID-19 impact on NO_2_ pollution” presents the NO_2_ variations observed before, during, and after the COVOD-19 lockdown for regions in Asia, Europe, North America, and South America dominated by anthropogenic emissions. The summary is given in “Conclusion”.

## Spaceborne NO_2_ measurements

### GOME-2

GOME-2 is a nadir-scanning ultraviolet, visible, and near-infrared spectrometer measuring the Earth’s backscattered radiance and extra-terrestrial solar irradiance in the spectral range between 240 and 790 nm. The first GOME-2 was launched in October 2006 aboard the EUMETSAT MetOp-A satellite, and a second GOME-2 was launched in September 2012 aboard MetOp-B (throughout this study referred to as GOME-2A and GOME-2B, respectively). The consistent long-term dataset is further extended by the third GOME-2 on the MetOp-C platform launched in November 2018. The Sun-synchronous polar orbit has a daily equator crossing time of $\sim $9:30 local time. The default swath width of GOME-2 is 1920 km, and the default ground pixel size is 80 km×40 km in the forward scan. A decreased swath of 960 km and an increased spatial resolution of 40 km×40 km are employed by GOME-2A in a tandem operation of MetOp-A and MetOp-B from July 2013 onwards. See Munro et al. (2016) for more details on instrument design and performance.

The operational GOME-2 NO_2_ products are generated using the GOME Data Processor (GDP) algorithm (Valks et al. 2011) and provided by DLR in the framework of EUMETSAT’s Satellite Application Facility on Atmospheric Composition Monitoring (AC SAF). Near-real-time, offline, and reprocessed GOME-2 level 2 and consolidated products are available via a dedicated FTP server and the EUMETSAT Data Centre (https://acsaf.org/). In the present study, the current operational product (Valks et al. 2020) is used.

### TROPOMI

TROPOMI is a push broom imaging spectrometer covering wavelength bands between the ultraviolet and the shortwave infrared, launched in October 2017 aboard the EU/ESA Sentinel-5 Precursor satellite. TROPOMI provides NO_2_ observations with a spatial resolution of 5.5 km×3.5 km at nadir (7 km×3.5 km before August 2019). The swath width is $\sim $2600 km in the direction across the track of the satellite that allows daily global coverage. In combination with the morning observations from GOME-2, the early afternoon measurements ($\sim $13:30 local time) from TROPOMI allow a better study of NO_2_ diurnal variations. For further details, see Veefkind et al. (2012) and Kleipool et al. (2018).

The TROPOMI NO_2_ retrieval algorithm used in this study is developed at DLR and has been used to analyze the effect of traffic emission on air quality in Germany (https://atmos.eoc.dlr.de/sveld/). The retrieval is based on an improved algorithm originally designed for GOME-2 (Liu et al. 2019; Liu et al. 2020) and adapted for TROPOMI measurements with optimization related to the specific instrumental aspects. The TROPOMI NO_2_ dataset used in the study is available upon request.

### Tropospheric NO_2_ column retrieval

The retrieval of tropospheric NO_2_ columns for the GOME-2 and TROPOMI instruments follows a classical three-step scheme. First, the slant columns (namely the concentrations integrated along the effective light path from the Sun through the atmosphere to the instrument) are derived from the measured (ir)radiances using the differential optical absorption spectroscopy (DOAS) method (Platt and Stutz 2008). Second, the stratospheric contribution is estimated and separated from the slant columns using a modified reference sector method (Valks et al. 2011; Beirle et al. 2016), which uses the measurements over regions with negligible tropospheric NO_2_ abundance. The modified reference sector method requires no additional model input and can be considered as a complement to the stratospheric correction based on data assimilation, as implemented in the operational TROPOMI product (van Geffen et al. 2020a). Third, the tropospheric NO_2_ vertical columns are converted from the tropospheric slant columns by an air mass factor (AMF) calculation. The presence of clouds is taken into account using cloud parameters based on the Optical Cloud Recognition Algorithm (OCRA) and Retrieval Of Cloud Information using Neural Networks (ROCINN) algorithms (Lutz et al. 2016; Loyola et al. 2018) with the advantages of operational implementation and routine validation (Compernolle et al. 2020; Lambert et al. 2020). The satellite data are filtered for clouds (cloud radiance fraction < 0.5 or cloud fraction < $\sim $0.3) to reduce retrieval errors. The retrieved GOME-2 and TROPOMI measurements are aggregated based on an area-weighted tessellation to resolutions of 0.1^∘^×0.1^∘^ and 0.025^∘^×0.025^∘^, respectively.

Based on ground-based multi-axis differential optical absorption spectroscopy (MAX-DOAS) measurements, the GOME-2 validation results are generally within the target accuracy of 30% for suburban and remote conditions (Pinardi et al. 2014; Pinardi et al. 2017). Larger underestimations are observed for polluted urban situations, because the large GOME-2 pixel size (80 km×40 km/40 km×40 km) is less representative of the local urban NO_2_ pattern sampled by the ground-based instrument (Pinardi et al. 2020). With a pixel size more representative of the NO_2_ fields on local and regional scales, the retrieved TROPOMI measurements agree with MAX-DOAS data at the suburban Xianghe site in China (Hendrick et al. 2014) with a correlation coefficient of 0.96 and a mean bias of − 2.1 × 10^15^ molec/cm^2^ or − 17.6% (Liu 2019, see Sect. 6.4 therein).

In comparison with the operational TROPOMI product (van Geffen et al. 2020a; van Geffen et al. 2020b), the retrieved TROPOMI tropospheric NO_2_ columns (Fig. S1) vary by 4 × 10^14^ molec/cm^2^ on average (Fig. S2) due to the difference in the stratosphere-troposphere separation method. From Fig. S2, larger increases by more than 1 × 10^15^ molec/cm^2^ are found mainly over polluted regions in winter as a result of the applications of different cloud parameters and different treatments of snow/ice scenarios in the AMF calculation (van der A et al. 2020).

### Trend, seasonal, and meteorological corrections

To consider the potential influences of long-term trends and seasonal cycles as well as short-term meteorological variations at a given location, the corrected tropospheric NO_2_ columns *V*_*corr*_ are calculated for GOME-2 and TROPOMI with a statistical model:

1 $$  V_{corr}=\frac{V+m_{trend}(t_{y},t_{m})}{f_{season}(t_{m}) \times f_{wind}(u(t),v(t))}. $$

For the observed time *t* (year *t*_*y*_, month *t*_*m*_, and day *t*_*d*_), the original tropospheric NO_2_ columns *V* are adjusted with a trend correction term *m*_*trend*_, a seasonal correction factor *f*_*season*_, and a wind correction factor *f*_*wind*_. The trend correction follows Bekbulat et al. (2020), who use the slope of historical data as the trend correction term for ground-based measurements and adjust the historical data to the 2020 reference. The seasonal correction and the wind correction apply the normalization method from Goldberg et al. (2020b), who modify the satellite observations to a reference with average seasonal and meteorological conditions.

The trend correction term *m*_*trend*_ is calculated for GOME-2A and GOME-2B as the slope of the linear regression line (Bekbulat et al. 2020) based on GOME-2A annual averages from 2007 to 2019. The assumption of linear trend has been widely used in previous works (Richter et al. 2005; Konovalov et al. 2010; Duncan et al. 2016; Georgoulias et al. 2019). To detect trend reversals in the $\sim $13-year time series, a method suggested by Cermak et al. (2010) is used to find the year when a reversal from negative to positive trends or from positive to negative trends happens. Identified by minimizing a change point score, a trend reversal is reported if the trend for the period before or after the reversal year is statistically significant at the 95% confidence level and the long-term average tropospheric NO_2_ columns is larger than 1 × 10^15^ molec/cm^2^ (Georgoulias et al. 2019). As shown in Fig. S3, extended regions over eastern China and parts of the North India Plain exhibit a reversal from positive to negative trends mostly in 2011 or 2012, in agreement with previous studies using satellite measurements or emission data (De Foy et al. 2016; van der A et al. 2017; Georgoulias et al. 2019). These reversals are partly explained by the implementation of clean technology (Bansal and Bandivadekar 2013; Liu et al. 2016), a stricter control of Chinese environmental regulations (CAAC 2013; Wu et al. 2017), and a slowdown in Indian economic development (Hilboll et al. 2017).

Figures 1 and 2 show the long-term trends of tropospheric NO_2_ columns measured by GOME-2A for eastern China (21^∘^N–41^∘^N, 110^∘^E–122^∘^E) and northern Italy (45^∘^N–46.5^∘^N, 7^∘^E–13^∘^E), including the slope and intercept of the linear regression analysis. For China, the annual trend correction is 0.69 × 10^15^ molec/cm^2^ per year for 2007–2011 and − 0.53 × 10^15^ molec/cm^2^ per year for 2011–2019, indicating changes of 8.3%/year and − 7.2%/year, respectively. The evaluations are confirmed by studies using OMI satellite measurements (Krotkov et al. 2016; Duncan et al. 2016; Georgoulias et al. 2020), who reported average changes of 8.4%/year before 2011 and − 7.0%/year after 2011 for eastern China, and studies using emission data (Liu et al. 2016; van der A et al. 2017), who estimated an increase of 9.1% and a decrease of 6.5%. For Italy, the annual trend correction is − 0.23 × 10^15^ molec/cm^2^ per year, representing a reduction of 4.3%/year of the tropospheric NO_2_ columns. Due to tightening vehicle emission standards (Euro 2007), a similar negative trend is detected by OMI (e.g., − 4.0%/year from Duncan et al. (2016)) and emission data (e.g. -5.2%/yr from Miyazaki et al. (2017)). Additional examples are gathered in Fig. S4 for large urban cities, where a negative trend is found for Los Angeles (− 3.5%/year) in the USA (33.5^∘^N–35.5^∘^N, 117.25^∘^W–119.25^∘^W), and positive trends are observed for New Delhi (1.2%/year) in India (27.6^∘^N–29.6^∘^N, 76.2^∘^E–78.2^∘^E) and Lima (2.8%/year) in Peru (11^∘^S–13^∘^S, 76^∘^W–78^∘^W).

The seasonal correction factor *f*_*season*_ is calculated for GOME-2A, GOME-2B, and TROPOMI based on the climatological seasonal variability (the monthly averages divided by the annual average), derived using NO_2_ observations from 2007 to 2019 for GOME-2A, 2013 to 2019 for GOME-2B, and 2018 to 2019 for TROPOMI, respectively. From the GOME-2A time series examples in Figs. 1 and 2 and the multiple-year monthly mean data over Asia, Europe, and North America in Fig. S5, the tropospheric NO_2_ columns are largest in winter, and the seasonal correction factors are $\sim $3–4 times higher in winter than in summer (Fig. S6), mainly due to the longer NO_2_ lifetime and higher emissions. As indicated by the EDGAR-HTAP_V2 emission data (https://edgar.jrc.ec.europa.eu/htap_v2/) in Fig. S7, the emissions from the residential sector, which is one of the main energy-related sources of NO_2_, increase for these northern mid-latitude regions in winter due to domestic heating.

The wind correction factor *f*_*wind*_ is derived using the eastward and northward wind components *u* and *v*, respectively, from the European Center for Medium range Weather Forecasting (ECMWF) ERA5 dataset (https://cds.climate.copernicus.eu/). The wind data at 10 m above the surface have a spatial resolution of 0.25^∘^×0.25^∘^ and a temporal resolution of 6 h. For different wind directions, wind speeds are averaged over 12 h prior to the satellite overpass time to approximately represent the effect of transport integrated over the lifetime of NO_2_ (Zhou et al. 2012). The 10 m data are representative of the wind fields within the lower boundary layer, particularly for regions with strong sources close to the surface (Hayn et al. 2009; Georgoulias et al. 2020). Influences of additional meteorological variables, such as precipitation, temperature, and solar irradiation, are partially considered by applying the corrections for season and wind (Zhou et al. 2012; Goldberg et al. 2020b). The cloud effects are not considered as the observations are filtered for clouds (see “Tropospheric NO_2_ column retrieval”).

**Fig. 3:**
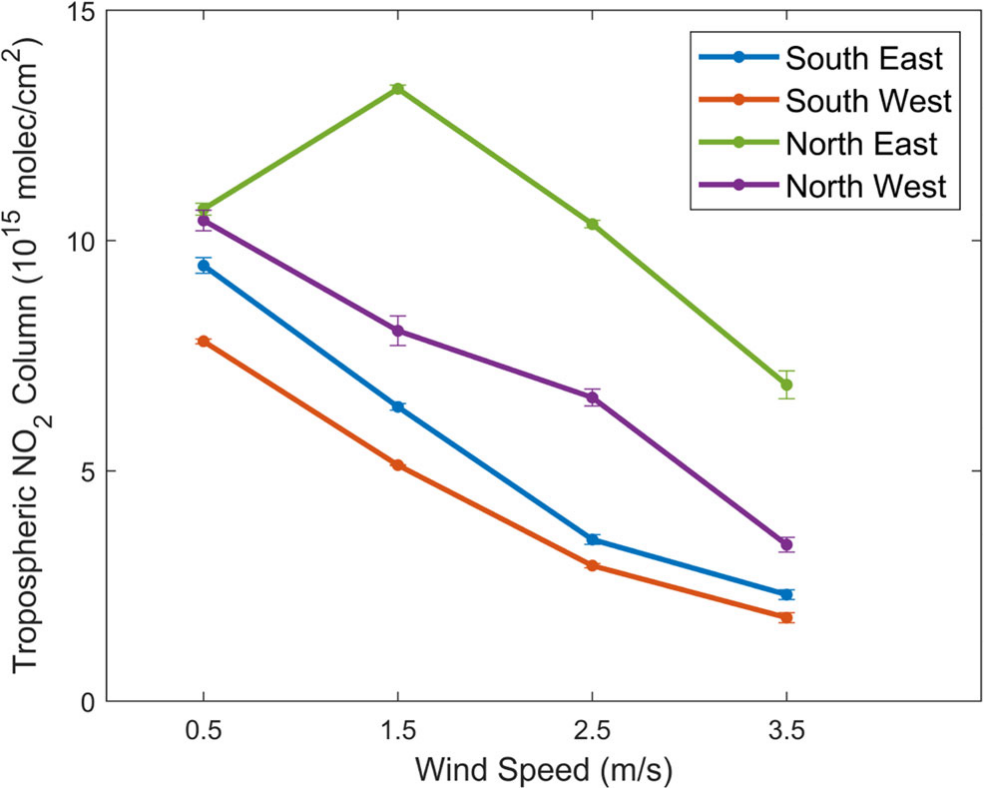
Variations in TROPOMI tropospheric NO_2_ columns for 2018–2019 as a function of wind speed for different wind directions over Los Angeles in the southwestern USA (34.0^∘^N–34.25^∘^N, 118.0^∘^W–118.25^∘^W). The error bar shows the standard error of the mean. The average wind speed is 1.6 m/s. The average tropospheric NO_2_ column is 7.1 × 10^15^ molec/cm^2^

Figure 3 shows the wind influences on the tropospheric NO_2_ columns measured by TROPOMI in 2018–2019 for Los Angeles located in the southwestern coast of the USA (34.0^∘^N–34.25^∘^N, 118.0^∘^W–118.25^∘^W). The TROPOMI tropospheric NO_2_ columns decrease by 33–76% when the wind speed increases from 0.5 to 3.5 m/s due to the faster dispersion away from the emission sources. For Los Angeles, the northeast wind (i.e., u < 0 and v < 0) yields the largest tropospheric NO_2_ columns with emission and transport from upwind, which can be 70% larger than the columns from other directions at the same wind speed, in agreement with estimations from the regional study from Goldberg et al. (2020b) using the operational TROPOMI data.

For each satellite pixel, *f*_*wind*_ is implemented following the normalization method from Goldberg et al. (2020b). First, the ERA wind data with a 0.25^∘^×0.25^∘^ resolution are bilinearly interpolated to 0.1^∘^×0.1^∘^ for GOME-2 and 0.025^∘^×0.025^∘^ for TROPOMI. Second, wind climatologies are derived using the historical wind information and tropospheric NO_2_ columns (2007–2019 for GOME-2A, 2013–2019 for GOME-2B, and 2018–2019 for TROPOMI), indicating the NO_2_ dependencies on wind speed and wind direction. Third, based on the wind climatologies, correction values for each grid pixel are determined by normalizing to a reference that is set to have an average wind speed. Lastly, the correction values are modelled by the linear regression, based on which *f*_*wind*_ is determined for the current ERA5 wind conditions.

**Fig. 4:**
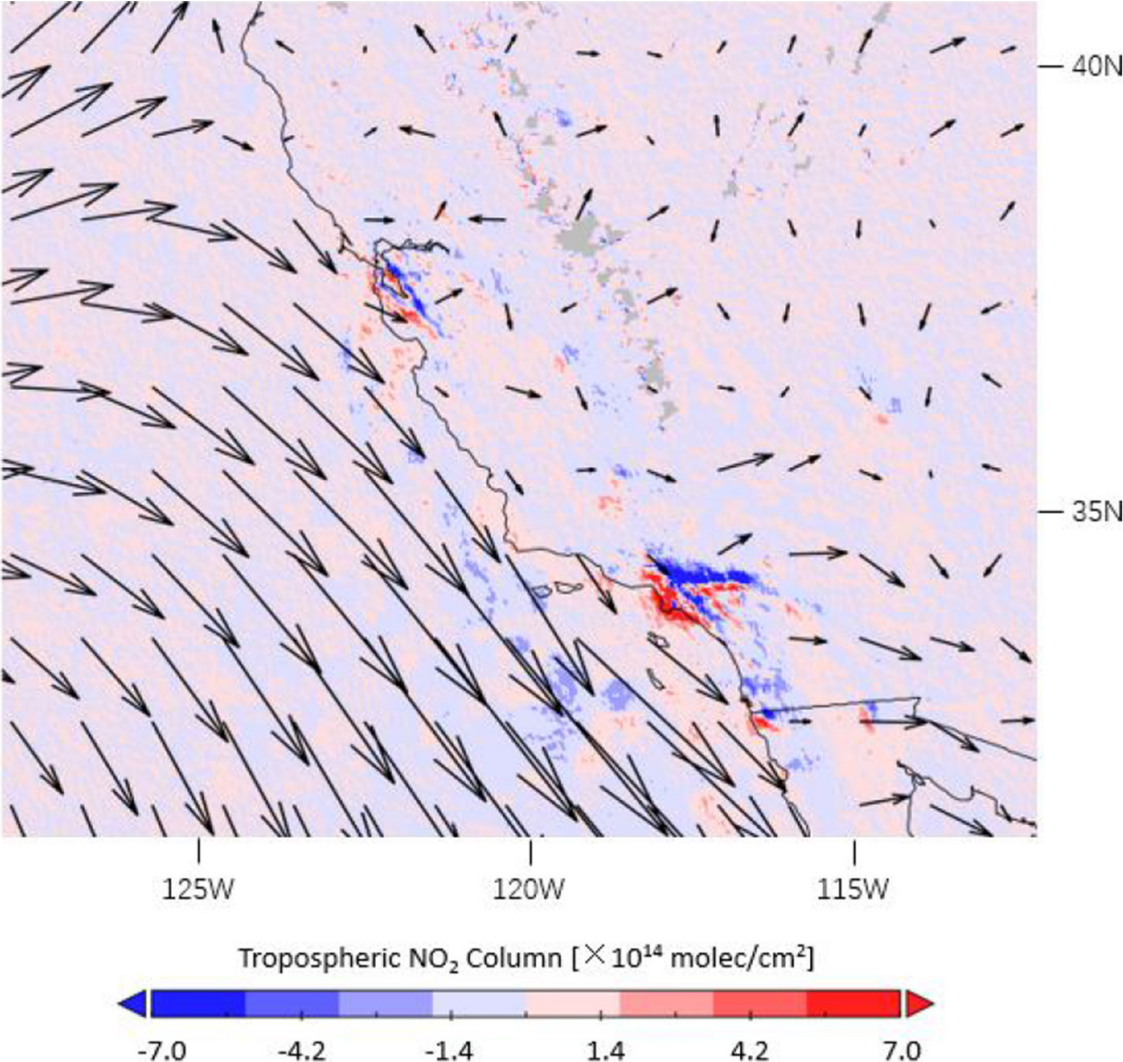
Average ERA5 wind directions and wind speeds as quiver plot and differences in tropospheric NO_2_ columns with and without the meteorological correction, as measured by TROPOMI over the southwestern USA in 16 March–15 April 2019

Figure 4 shows the average wind directions and wind speeds in 16 March–15 April 2019 and the effect of applying the meteorological correction for TROPOMI tropospheric NO_2_ columns over the southwestern USA area. For Californian coastal cities with a mountainous terrain, such as Los Angeles and San Francisco, taking account of the meteorological condition (predominant west winds) affects the NO_2_ levels by up to 1 × 10^15^ molec/cm^2^ (20%), mainly by reducing the NO_2_ underestimations for upwind regions and reducing the overestimations for downwind regions.

## COVID-19 impact on NO_2_ pollution

### China

China, where COVID-19 was first identified, was also the first country to impose the lockdown restrictions, starting from Wuhan and other cities in the Hubei region on 23 January 2020 to quarantine the center of COVID-19 outbreak. Similar measures have been imposed across China as of mid-March 2020. With the efforts mostly based on strict containment measures, the first epidemic wave has been under control by early April 2020, when the lockdowns ended or were largely relaxed.

**Fig. 5:**
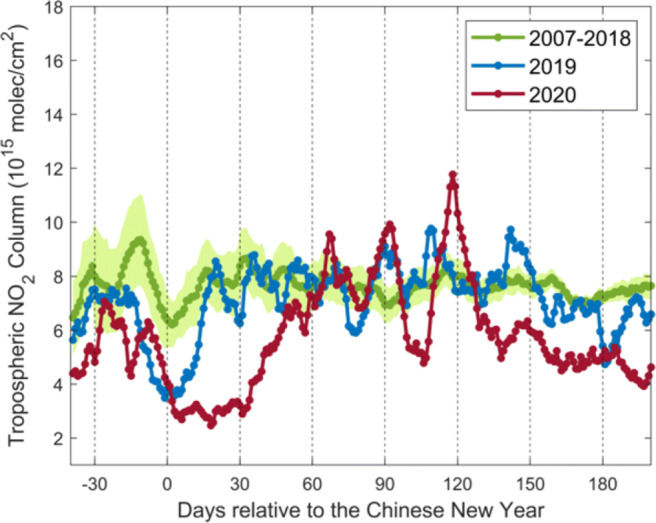
Daily variations in 10-day moving averages of the corrected GOME-2A/B tropospheric NO_2_ columns over eastern China (21^∘^N–41^∘^N, 110^∘^E–122^∘^E) for 2007–2018 (green), 2019 (blue), and 2020 (red). Green shading shows standard error of the mean for 2007–2018. The 2020 COVID-19 pandemic lockdown starts on 23 January. The 2020 Chinese New Year falls on 25 January. The yearly varying dates of the Chinese New Year are considered

Figure 5 shows the tropospheric NO_2_ daily variations in 10-day moving averages over eastern China (21^∘^N–41^∘^N, 110^∘^E–122^∘^E) for 2020 and historical data from GOME-2A/B. Data are corrected for trend, season, and meteorology. In comparison with the uncorrected values in Fig. S8, the winter values in Fig. 5 are uniformly lower after accounting for the seasonal influence, and the summer values are higher. The meteorological correction affects the GOME-2 tropospheric NO_2_ columns by up to 8.5% during lockdown in Fig. S9. Smaller positive corrections factors in 2020 during lockdown indicate less favorable conditions for low NO_2_ as compared to 2019, in agreement with Liu et al. (2020a).

The analysis of the COVID-19 lockdown impact is complicated by the coincidence of the 7-day Chinese New Year holidays. Consistent with the historical data, the tropospheric NO_2_ columns in 2020 decrease by a factor of 2 before the New Year. The columns, however, do not increase back to the normal level after the holiday as the historical data mainly resulted from the lockdown measures, confirming previous findings using OMI data (Bauwens et al. 2020; Huang and Sun 2020). Due to the gradual recovery of social and economic activities, the columns start to rebound 1 month after the New Year (late February) and return to the normal level as previous years by early April with short-term variations partly related to observational errors. Higher NO_2_ levels are found during April and May 2020 due to the increased emissions from energy consumption and road transport (Zheng et al. 2020), consistent with regional studies using ground-based monitoring data (Wang et al. 2020; Silver et al. 2020; Lauri 2020) and emission data (Zheng et al. 2020a). Since the 2020 NO_2_ data are lower by 31.8% than previous years before the lockdown period and 16.3% after the lockdown period, likely due to the pollution control policies (Liu et al. 2016; Wu et al. 2017), the lockdown effect over eastern China is estimated to induce a NO_2_ reduction of 30% on average.

Consistent daily variations of the retrieved NO_2_ amounts are found between GOME-2 and TROPOMI (Fig. S10 - S12). For the urban and suburban Chinese regions, the NO_2_ measurements from GOME-2 are generally larger than TROPOMI due to the diurnal cycle of NO_2_, which is a function of diurnal variability in emissions, photochemistry, and boundary layer height (Penn and Holloway 2020). Attributed in part to emissions from commuter traffic, which peak in the morning and evening (Bower et al. 1991; Ketzel et al. 2003; Harley et al. 2005), the GOME-2 overpass could capture morning maximum NO_2_ columns (Fishman et al. 2008; Penn and Holloway 2020). In addition, the GOME-2 measurements are generally noisier as compared to TROPOMI results because of instrument degradation effects (Munro et al. 2016).

**Fig. 6:**
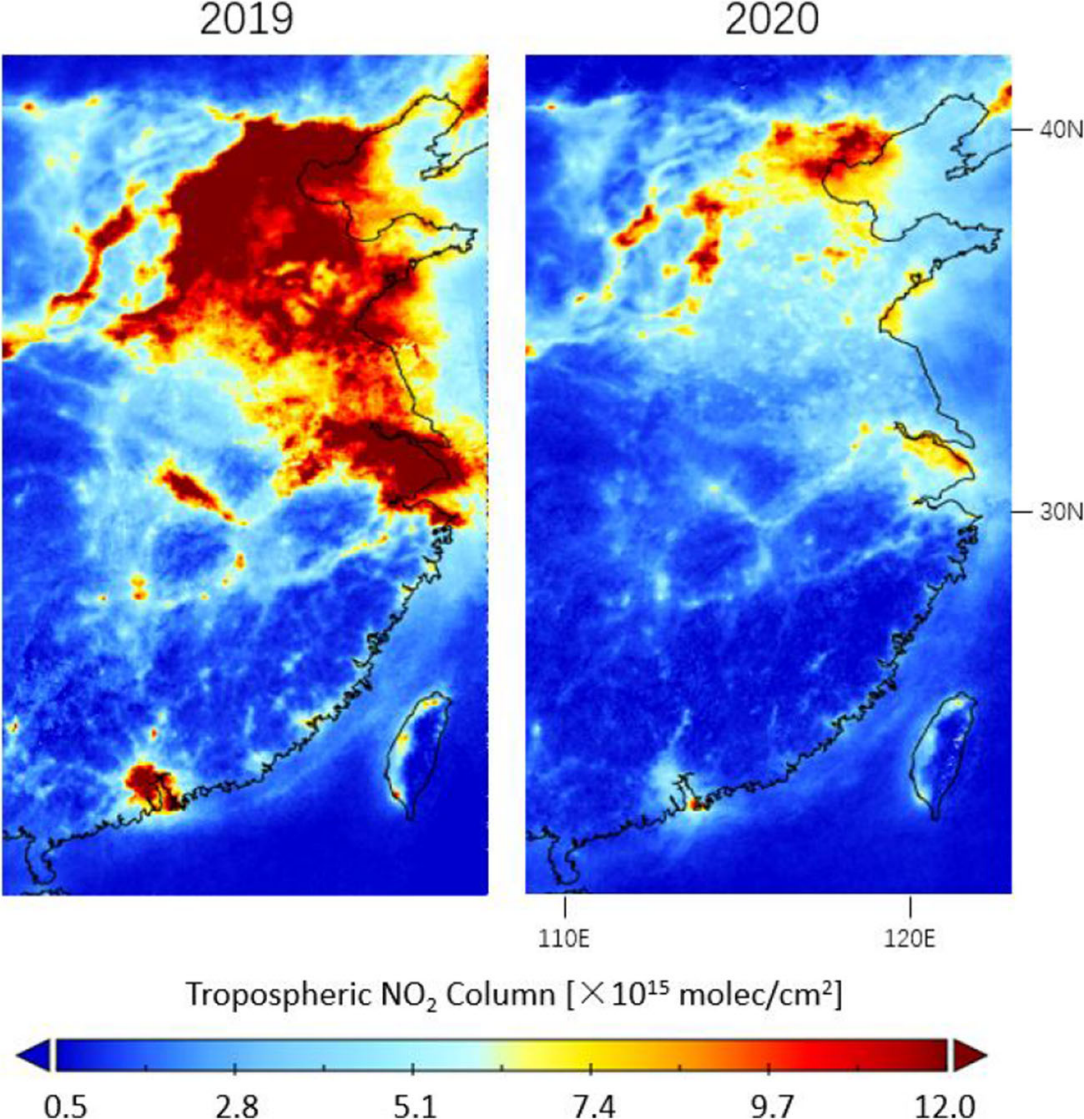
Averages of the corrected tropospheric NO_2_ columns measured by TROPOMI over eastern China during lockdown in 23 January–22 March in 2020 and comparison with columns in the same time period in 2019

Figure 6 shows the corrected TROPOMI tropospheric NO_2_ over eastern China during the 2020 COVID-19 lockdown and the comparisons with columns in the same time periods in 2019. During the lockdown period, the TROPOMI NO_2_ declines across China, including the industrial regions and economic zones in the North, the major highways in the Center, and the shipping routes in the South.

**Fig. 7:**
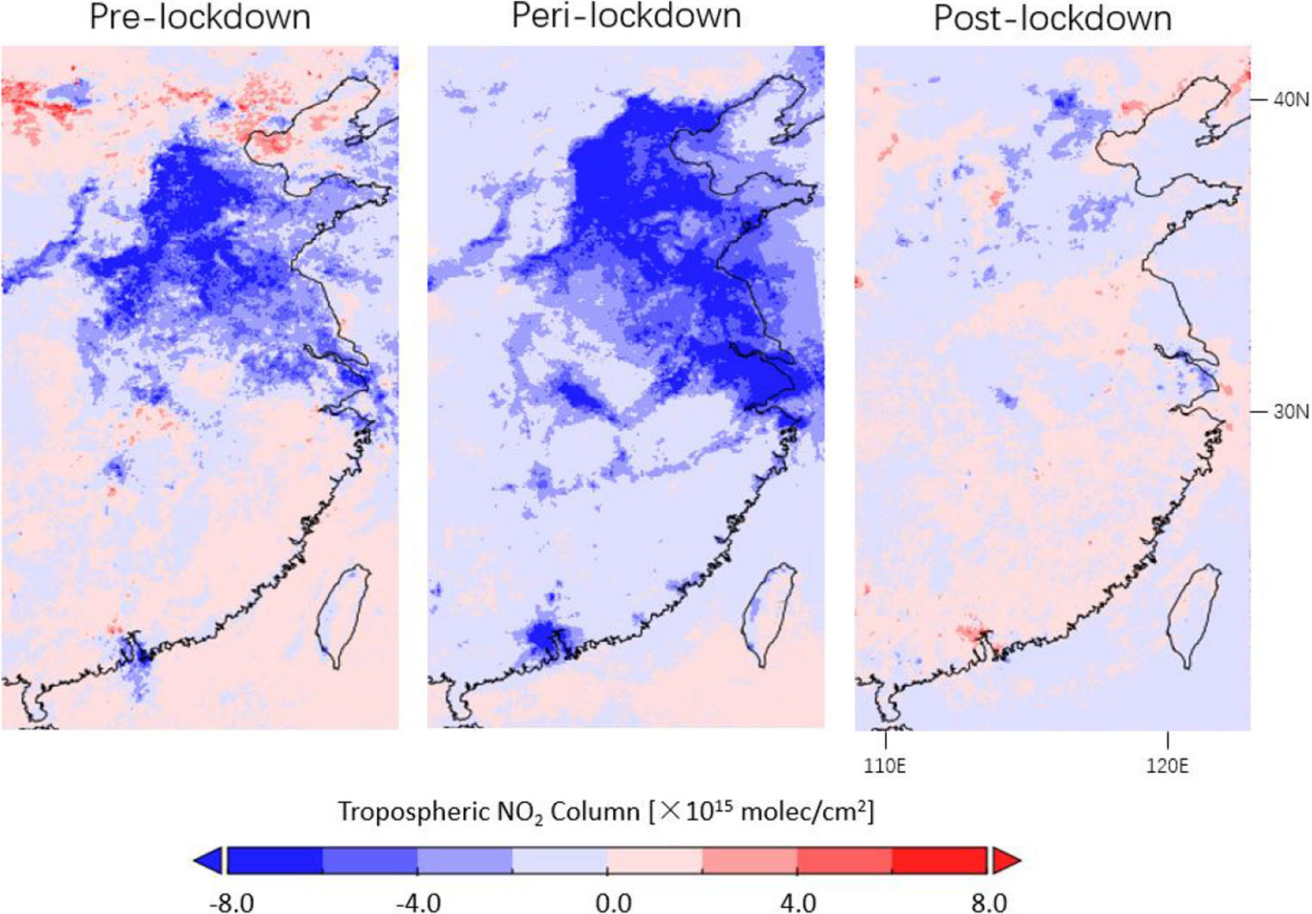
Differences in corrected TROPOMI tropospheric NO_2_ columns between 2020 and 2019 observed before (23 November of the previous year–22 January), during (23 January–22 March), and after (23 March–22 May) lockdown over eastern China

Figure 7 presents the TROPOMI NO_2_ differences between 2020 and 2019 over eastern China for the pre-lockdown period (23 November of the previous year–22 January), the peri-lockdown period (23 January–22 March), and the post-lockdown period (23 March–22 May). Table 1 quantifies the impact of the lockdown on corrected TROPOMI tropospheric NO_2_ columns at selected Chinese cities. The populated cities show strong reductions of tropospheric NO_2_ columns during lockdown, likely due to a combination of general improvements in air quality (reductions by up to 33%) and COVID-19 lockdown impact (further reductions by 27–48%). The estimated lockdown impact is consistent with Liu et al. (2020a), who observed a lockdown-related decrease of the operational TROPOMI NO_2_ data by 21 ± 5% and concluded that the actual emission reduction is likely larger than the observed decrease due to the meteorological influence.

**Table 1:** Relative differences in the corrected tropospheric NO_2_ columns (%) between 2020 and 2019 observed before, during, and after the COVID-19 lockdown for selected cities in Asia, Europe, North America, and South America

Name	Location	Pre-lockdown	Peri-lockdown	Post-lockdown
		23 Nov(last year)–22 Jan	23 Jan–22 Mar	23 Mar–22 May
Beijing, China	39.92^∘^N, 116.42^∘^E	− 4.43	− 45.7	− 28.0
Shanghai, China	31.17^∘^N, 121.47^∘^E	− 33.1	− 60.5	− 15.0
Wuhan, China	30.58^∘^N, 114.28^∘^E	− 22.4	− 70.8	− 26.1
		16 Jan–15 Mar	16 Mar–15 May	16 May–15 Jul
Barcelona, Spain	41.38^∘^N, 2.15^∘^E	− 13.1	− 62.4	− 31.3
Lisbon, Portugal	38.73^∘^N, 9.15^∘^W	− 22.7	− 49.6	− 18.4
Madrid, Spain	40.43^∘^N, 3.70^∘^W	− 21.4	− 59.9	− 37.1
Milan, Italy	45.45^∘^N, 9.17^∘^E	− 30.2	− 50.3	− 26.5
Rome, Italy	41.90^∘^N, 12.45^∘^E	− 12.9	− 44.5	− 28.4
		25 Jan–24 Mar	25 Mar–24 Jun	25 Jun–24 Jul
New Delhi, India	28.61^∘^N, 77.21^∘^E	− 14.8	− 42.8	− 6.49
Mumbai, India	19.00^∘^N, 72.80^∘^E	1.45	− 41.3	− 19.5
Waidhan, India	24.11^∘^N, 82.65^∘^E	− 15.9	− 10.1	− 24.3
		16 Jan–15 Mar	16 Mar–15 May	16 May–15 Jul
Los Angeles, USA	34.05^∘^N, 118.25^∘^W	4.81	− 33.0	− 29.6
New York, USA	40.78^∘^N, 73.97^∘^W	− 16.4	− 37.3	− 23.9
Philadelphia, USA	39.95^∘^N, 75.17^∘^W	− 20.8	− 28.2	− 23.5
San Francisco, USA	37.78^∘^N, 122.43^∘^W	11.7	− 36.7	− 29.6
Washington DC, USA	38.88^∘^N, 77.03^∘^W	− 23.5	− 31.3	− 27.3
		16 Jan–15 Mar	16 Mar–15 May	16 May–15 Jul
Buenos Aires, Argentina	34.58^∘^S, 58.37^∘^W	13.0	− 26.1	− 4.36
Guayaquil, Ecuador	2.17^∘^S, 79.93^∘^W	− 11.4	− 45.1	− 5.19
Lima, Peru	12.00^∘^S, 77.03^∘^W	7.22	− 74.7	− 51.4
Santiago, Chile	33.47^∘^S, 70.7^∘^5W	− 3.34	− 25.7	− 29.7
Sao Paulo, Brazil	23.52^∘^S, 46.52^∘^W	34.8	− 24.9	− 20.1

Average tropospheric NO_2_ columns are calculated using TROPOMI data within a 0.5^∘^×0.5^∘^ box around the city centers (latitudes and longitudes given in the table)

### Southern Europe

As one of the first European countries hit hard by the COVID-19 pandemic, Italy imposed initial lockdown on 21 February 2020 in the most affected Lombardy region. The lockdown zone was extended to the northern provinces on 8 March and to the whole country on 9 March, making Italy the first European country to implement a nationwide lockdown. The lockdown restrictions were partially lifted from 4 May and further relaxed from 1 June.

**Fig. 8:**
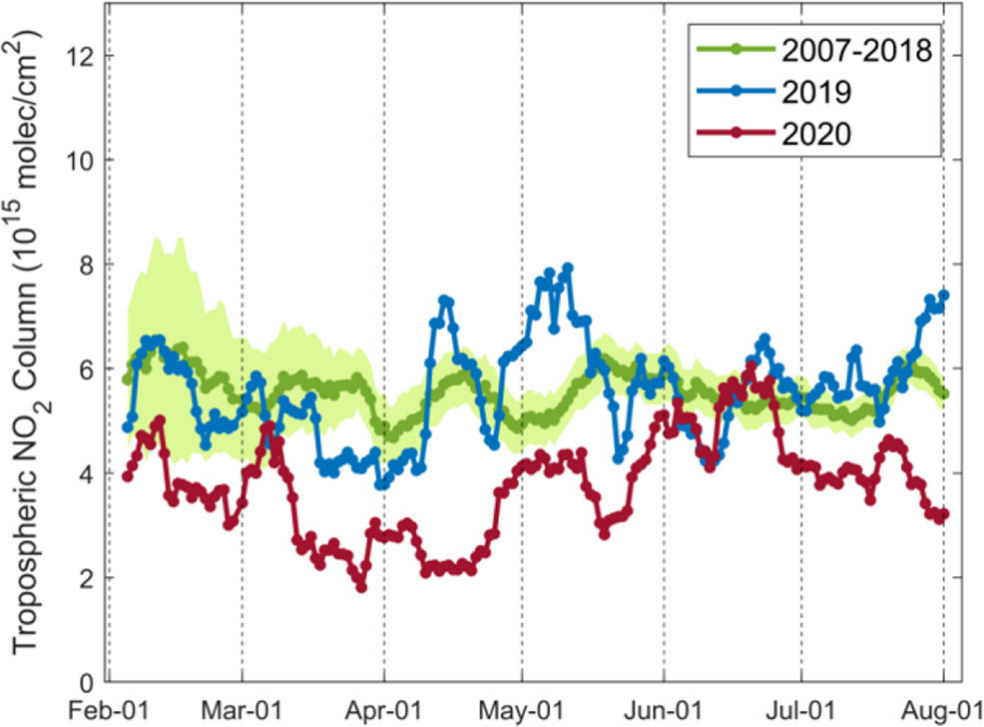
Daily variations in 10-day moving averages of the corrected GOME-2A/B tropospheric NO_2_ columns over northern Italy (45^∘^N–46.5^∘^N, 7^∘^E–13^∘^E) for 2007–2018 (green), 2019 (blue), and 2020 (red). Green shading shows standard error of the mean for 2007–2018. The COVID-19 pandemic lockdown starts on 8 March 2020

From Fig. 8, the corrected tropospheric NO_2_ columns from GOME-2A/B decrease by $\sim $20% for northern Italy before the lockdown compared to previous years, mainly caused by the emission control of the road transport and by the industrial combustion modification in Europe (Curier et al. 2014; Duncan et al. 2016; EEA 2019). The NO_2_ columns decline by 51.7% on average during the lockdown period in March–May and return to the level $\sim $20% lower compared to historical data in early June after the lockdown was eased. Considering the decrease before and after lockdown, the lockdown restriction measures likely contribute to an average 30% decline of the NO_2_ concentration for northern Italy.

Following Italy, European countries started to impose restrictions from mid-March with different severity and timing. Countries like Italy and Spain imposed strict lockdowns in response to the strong increase of infections, while others such as Germany and the Netherlands enacted relatively more relaxed measures. Countries like Portugal and Greece enforced proactive measures when cases were low, while others including France and the UK took longer before imposing strict lockdowns.

**Fig. 9:**
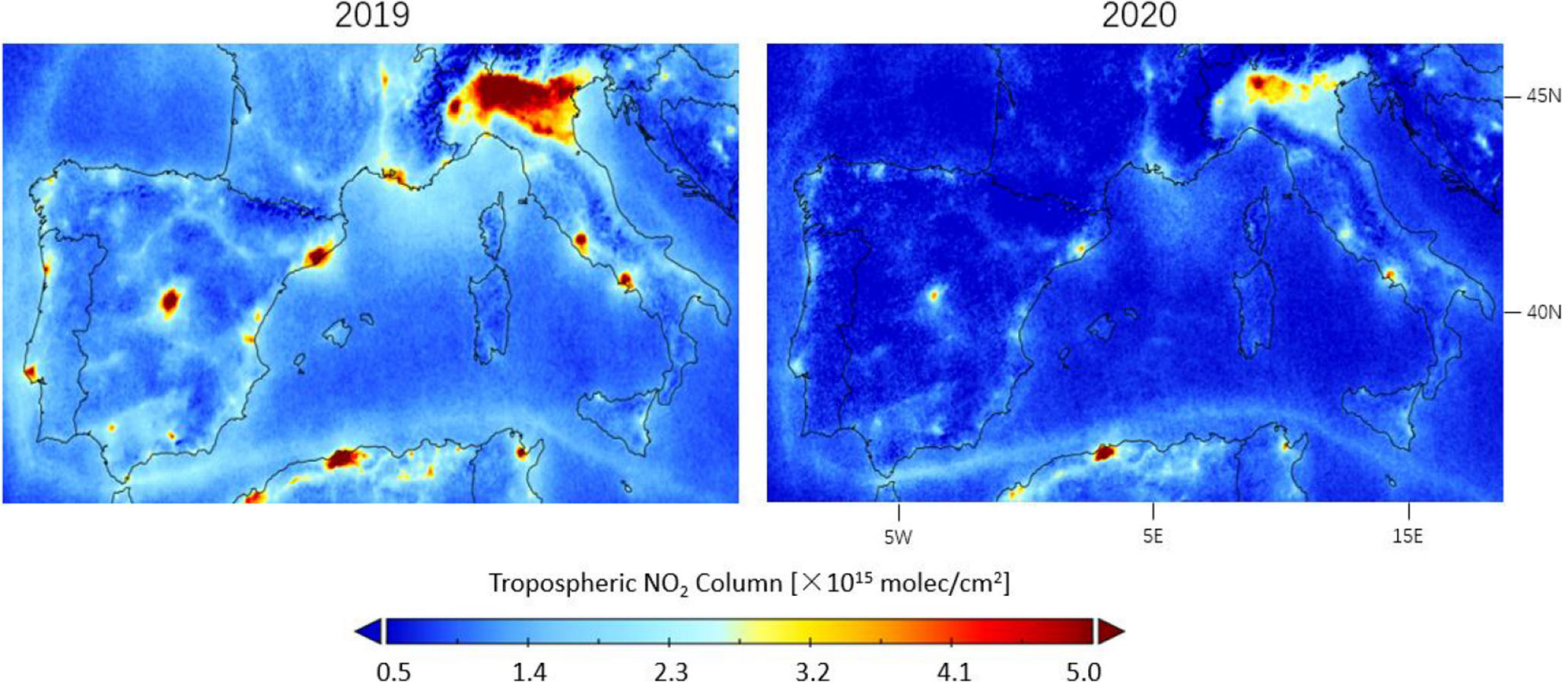
Averages of the corrected tropospheric NO_2_ columns measured by TROPOMI over southern Europe during lockdown in 16 March–15 May in 2020 and comparison with columns in the same time period in 2019

**Fig. 10:**
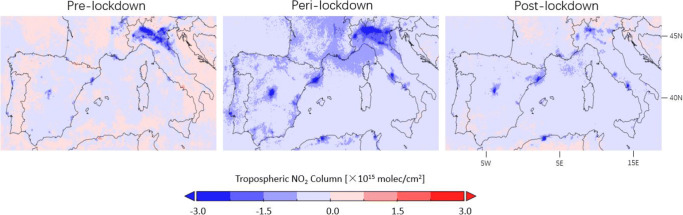
Differences in corrected TROPOMI tropospheric NO_2_ columns between 2020 and 2019 observed before (16 January–15 March), during (16 March–15 May), and after (16 May–15 July) lockdown over southern Europe

For countries with strict and rapid lockdown measures in southern Europe, the drop in road traffic and industrial activities during the lockdown period leads to visible decreases of NO_2_ levels in most cities and highways in Figs. 9 and 10. The observed tropospheric NO_2_ columns in Table 1 are 12.9–30.2% lower than the previous year before the implementation of the lockdown measures, 44.5–62.4% lower during lockdown, and 18.4–37.1% lower after lockdown, indicating that the lockdown effect contributes to a $\sim $30% drop in NO_2_ over southern Europe.

### India

India ordered a public curfew in response to the COVID-19 outbreak on 22 March 2020, followed by a nationwide lockdown affecting 1.3 billion people on 25 March 2020. This large lockdown was extended to 30 June for containment zones and was eased in a phased manner in other zones from 8 Jun.

**Fig. 11:**
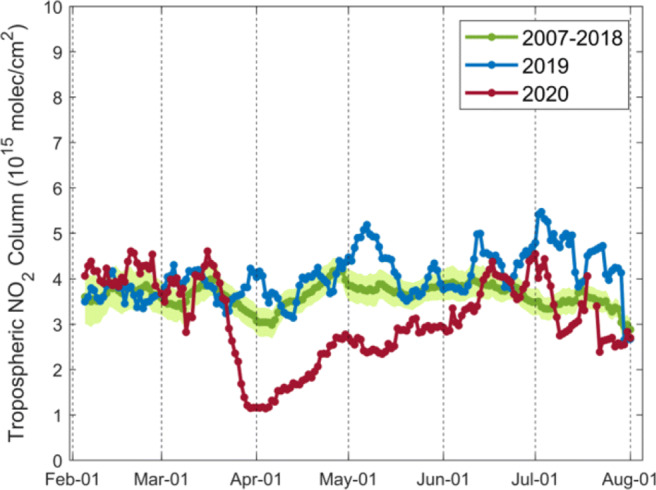
Daily variations in 10-day moving averages of the corrected GOME-2A/B tropospheric NO_2_ columns over New Delhi in northern India (27.6^∘^N–29.6^∘^N, 76.2^∘^E–78.2^∘^E) for 2007–2018 (green), 2019 (blue), and 2020 (red). Green shading shows standard error of the mean for 2007–2018. The COVID-19 pandemic lockdown starts on 25 March 2020

From Fig. 11, since the start of initial curfews and national restrictions in late March, the corrected GOME-2 tropospheric NO_2_ columns decrease by a factor of 3 by early April over the New Delhi region and remain low until June, not only in comparison with values before the lockdown but also compared to an identical period in the historical data. The declines for New Delhi during the lockdown period are up to 65.2% compared to previous years. Good agreement is observed between GOME-2 and TROPOMI data (Fig. S11).

**Fig. 12:**
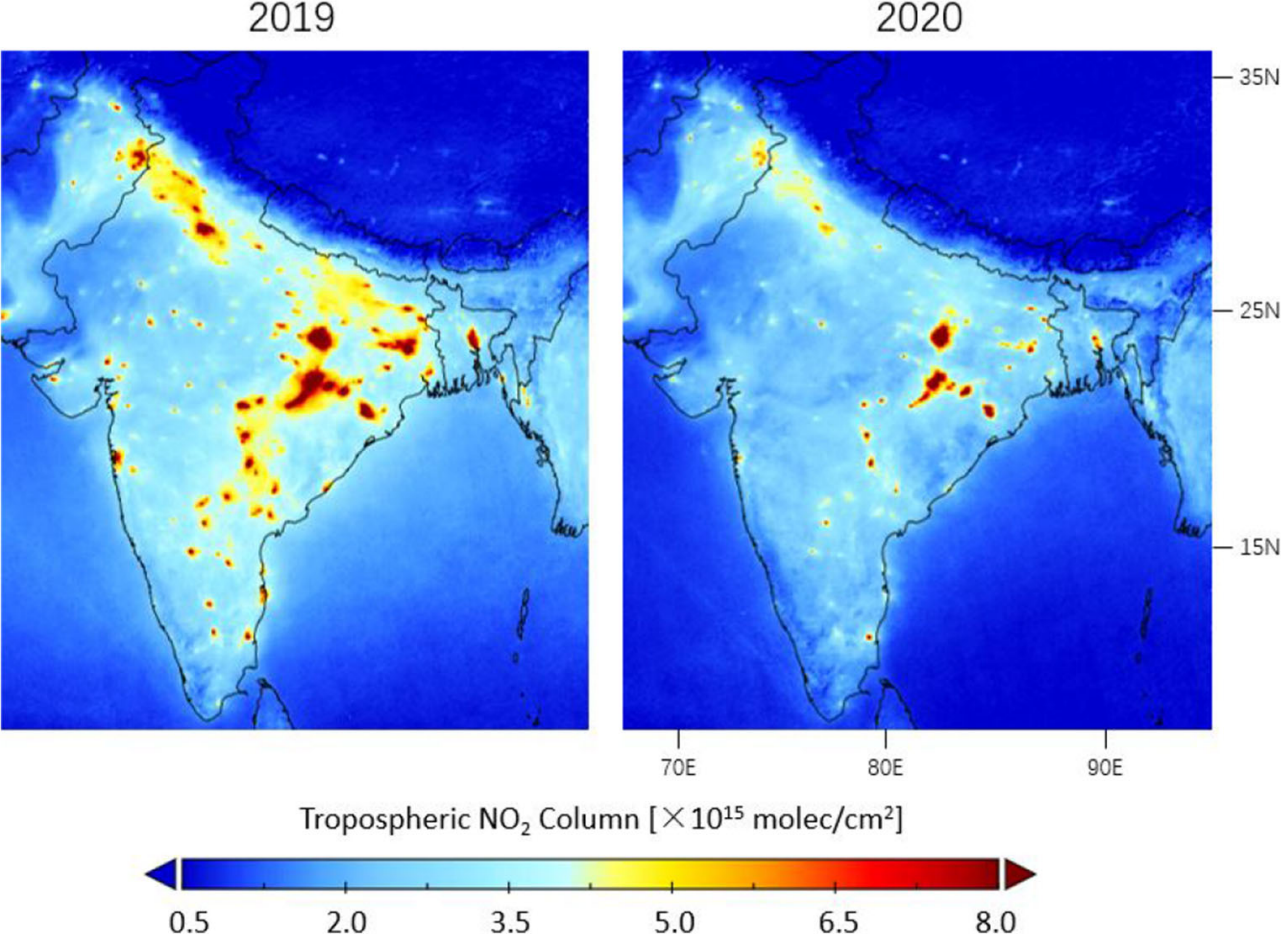
Averages of the corrected tropospheric NO_2_ columns measured by TROPOMI over India during lockdown in 25 March–24 June in 2020 and comparison with columns in the same time period in 2019

**Fig. 13:**
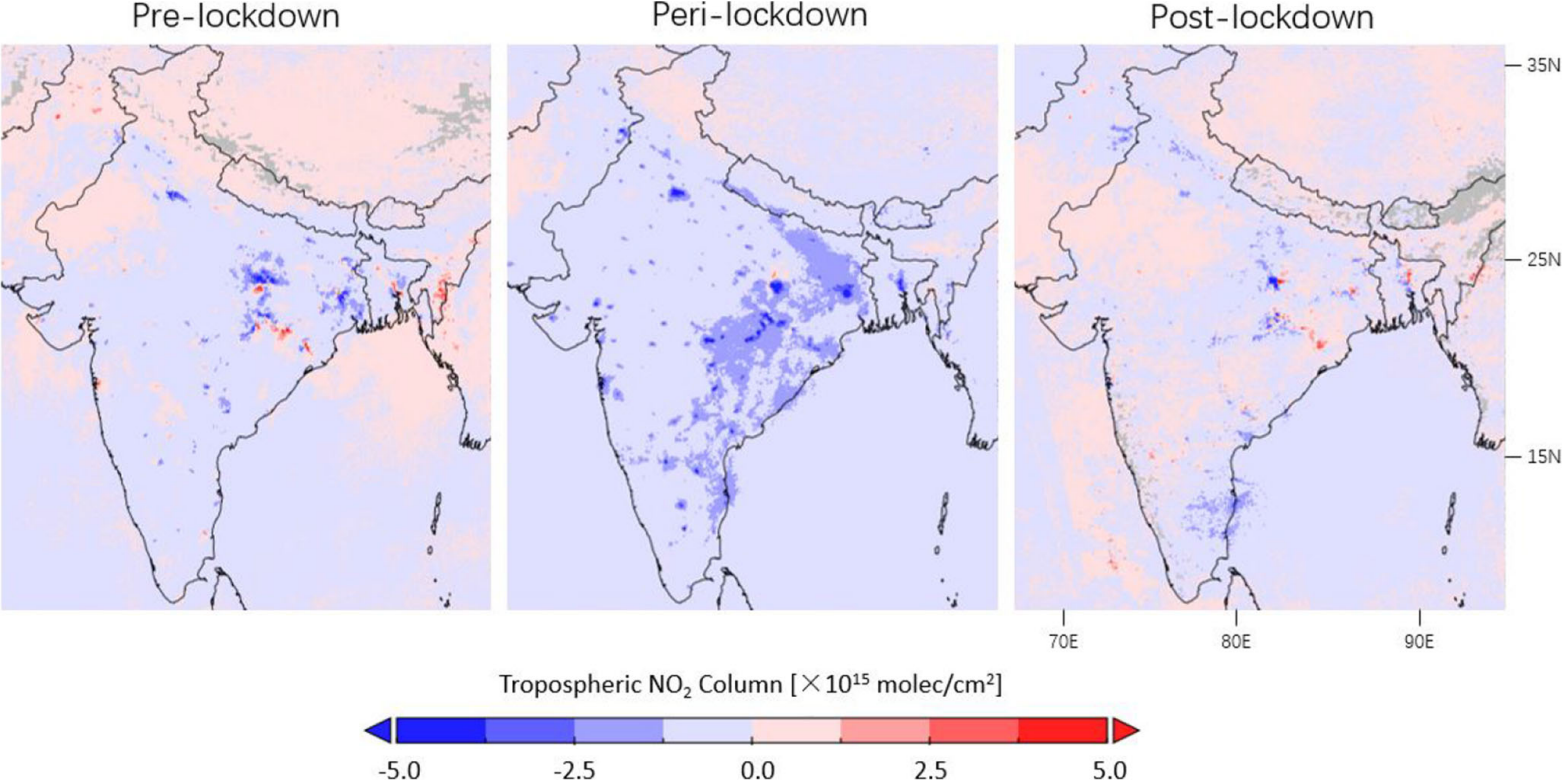
Differences in corrected TROPOMI tropospheric NO_2_ columns between 2020 and 2019 observed before (25 January–24 March), during (25 March–24 June), and after (25 June–24 July) lockdown over India

From Figs. 12 and 13, lockdown-related declines of TROPOMI tropospheric NO_2_ columns are observed for the Indo-Gangetic Plain in the North with a large population as well as the Chhattisgarh state in the Center and the Tamil Nadu state in the South with electricity production activities (Hilboll et al. 2017). From Table 1, the NO_2_ values decrease by 42% on average for populated cities such as New Delhi and Mumbai for the lockdown period compared to the same time in 2019, which is 35.4% lower than the pre-lockdown drops. For the Waidhan City with the largest Indian power station (the Vindhyachal Super Thermal Power Station), lockdown-related variations of no more than 14.2% are found in the TROPOMI dataset due to the continuous operations to procure coal-powered energy, an essential commodity during the lockdown period (Sharma et al. 2020; Mahato et al. 2020).

### The USA

The USA local and statewide restriction measures first began to come into effect from mid-March 2020 in affected areas like California. The Californian lockdown started first on 12 March to limit non-essential gatherings and extended to the entire state on 19 March. The restrictions were initially lifted from 8 May and further relaxed from 5 June. As of July 2020, however, California re-imposed the lockdown measures, when the highest number of confirmed infections in the USA was reported.

**Fig. 14:**
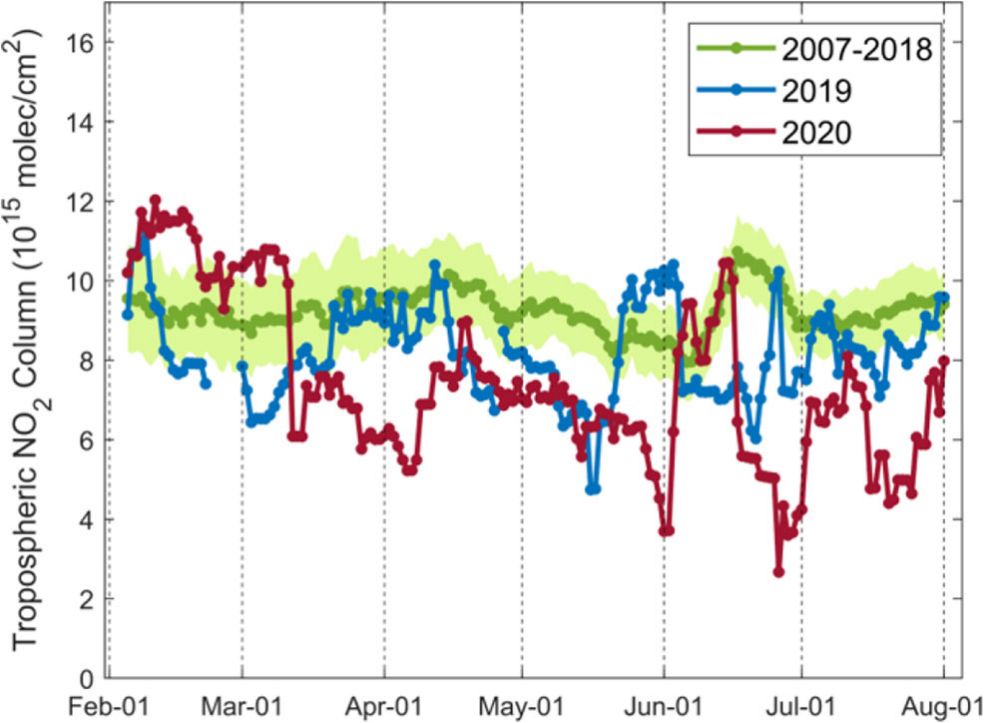
Daily variations in 10-day moving averages of the corrected GOME-2A/B tropospheric NO_2_ columns over Los Angeles in the southwestern USA (33.5^∘^N–35.5^∘^N, 117.25^∘^W–119.25^∘^W) for 2007–2018 (green), 2019 (blue), and 2020 (red). Green shading shows standard error of the mean for 2007–2018. The COVID-19 pandemic lockdown starts on 12 March 2020

The corrected NO_2_ concentrations from GOME-2 decline by 20% on 12 March for Los Angeles in Fig. 14 as the initial COVID-19 measures were adopted. Compared to the historical data, the NO_2_ concentrations are decreased by 31.1% on average during the first month of lockdown and 26.0% during the second month. The (much-)above-average to record precipitation in 2020 (https://www.ncdc.noaa.gov/sotc/national/202003) can contribute to the NO_2_ variations, but the impact is expected to be partially corrected by the wind correction introduced in “Trend, seasonal, and meteorological corrections”. In early June, the strong increases of tropospheric NO_2_ columns can be explained by the presence of a number of bush fires (https://www.lafd.org/alerts). Afterwards the NO_2_ levels remain low compared to the historical data, because the lockdown measures were re-imposed due to a significant increase of infection cases.

Similar variations are observed for TROPOMI measurements in Fig. S11. However, as compared to the relatively large regions with high pollution levels in “China”-India, Los Angeles shows less pronounced agreement between GOME-2 and TROPOMI data due to heterogeneous topography and isolation from urban agglomeration. In addition, a stronger dependency of local NO_2_ amount on wind fields is observed for TROPOMI (Fig. S12) than GOME-2 (Fig. S9). For Los Angeles, GOME-2 averages the high concentrations of the plume with the lower surrounding concentrations over a larger pixel size.

**Fig. 15:**
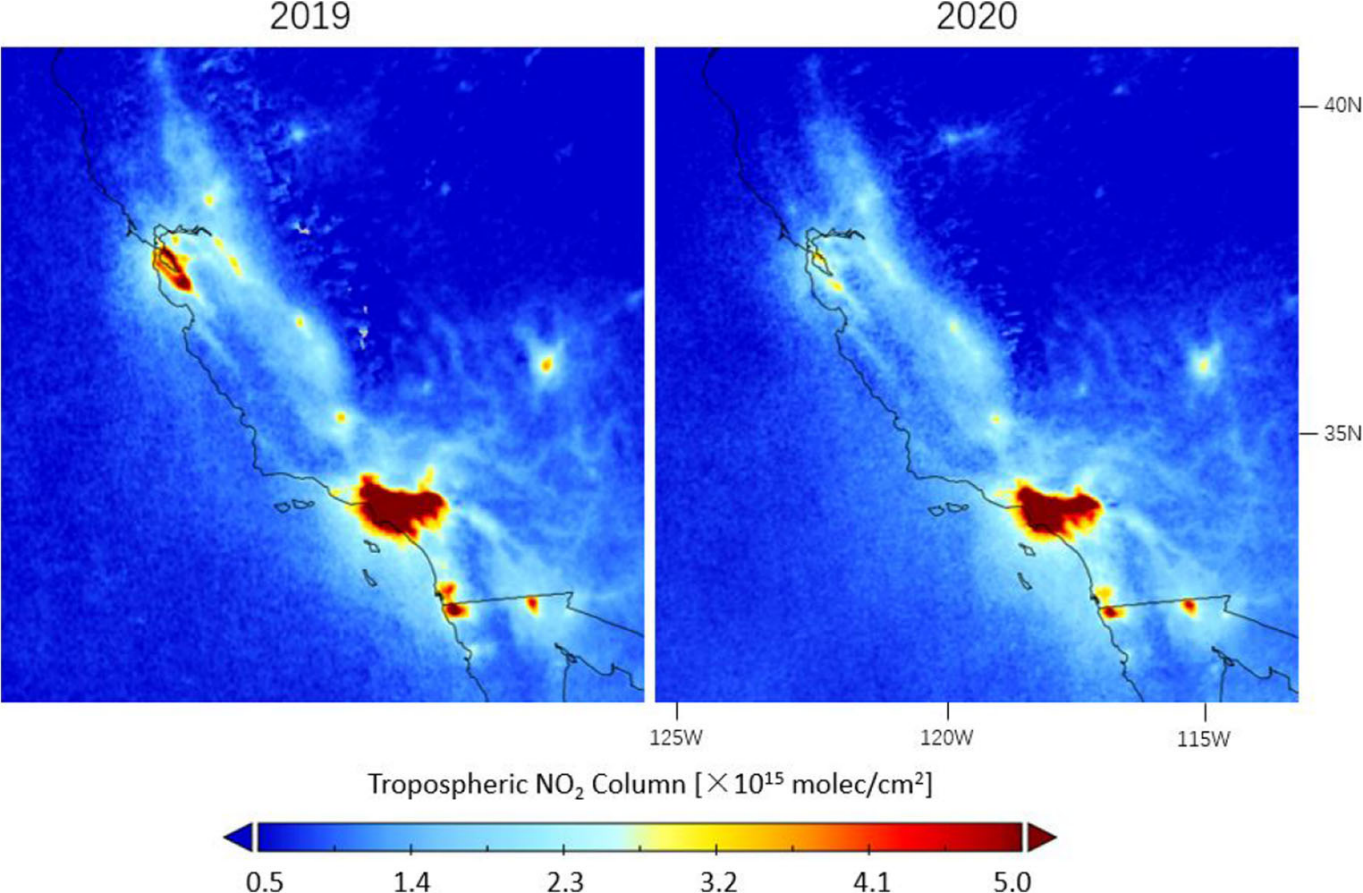
Averages of the corrected tropospheric NO_2_ columns measured by TROPOMI over southwestern USA during lockdown in 16 March–15 May in 2020 and comparison with columns in the same time period in 2019

**Fig. 16:**
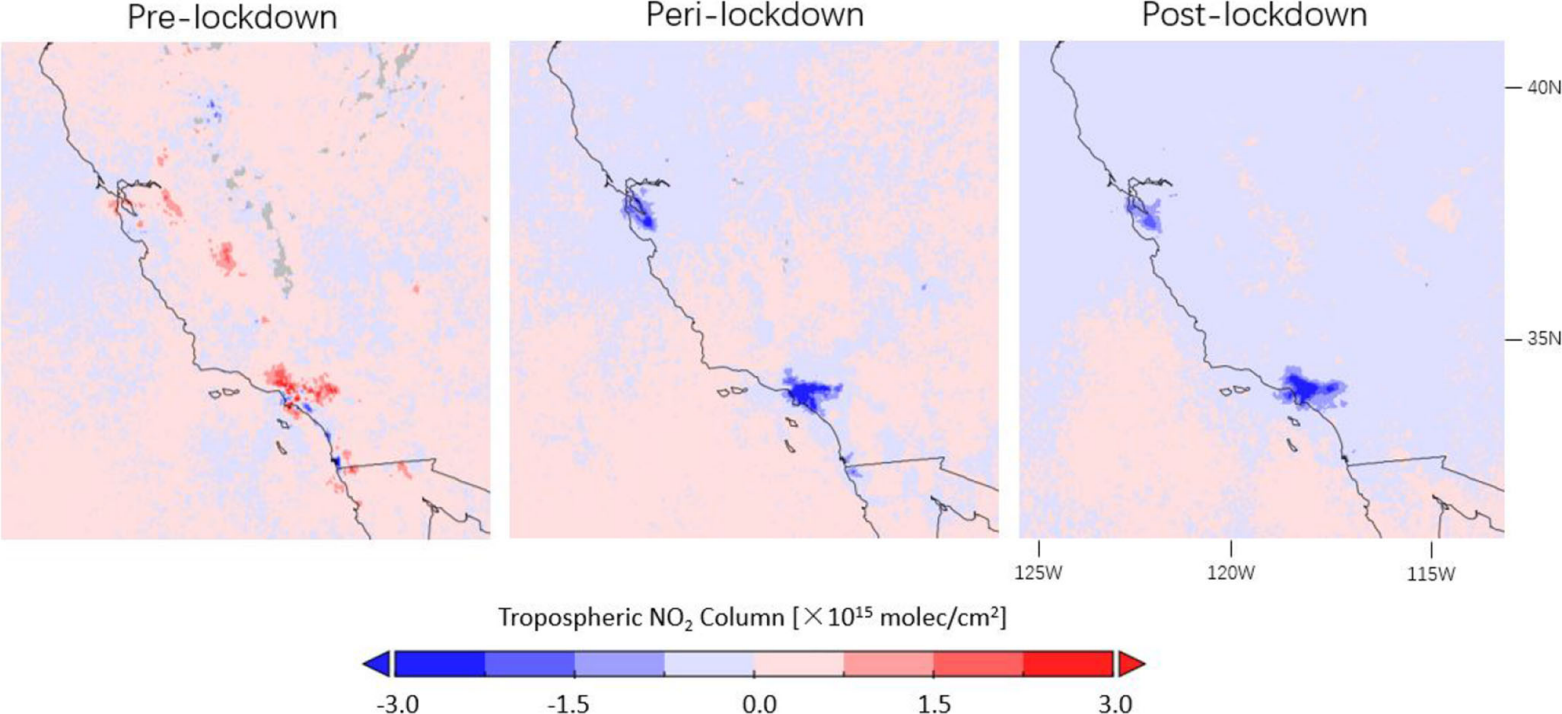
Differences in corrected TROPOMI tropospheric NO_2_ columns between 2020 and 2019 observed before (16 January–15 March), during (16 March–15 May), and after (16 May–15 July) lockdown over southwestern US

The lockdown causes decreases of TROPOMI NO_2_ levels for major cities in California’s Central Valley, the San Francisco Bay Area, and the Greater Los Angeles Area as compared to 2019 in Figs. 15 and 16. The mean decline observed during the lockdown period for California is 34.9% in Table 1, consistent with the estimations of 32.5 to 40.7% from Goldberg et al. (2020b) using the operational TROPOMI product and accounting for the meteorological effect. The NO_2_ columns recover only slightly by 5.2% between the peri- and post-lockdown periods, which can be related to the implementation of the re-lockdown, requiring future observations for a robust analysis. In comparison with California, the lockdown-related NO_2_ variations are less significant in the eastern USA in Table 1. The NO_2_ drops due to COVID-19 precautions are estimated to range between 7.4 and 17.8% considering the decline in the pre-lockdown period, and the NO_2_ rebounds range between 4.0 and 10.3%.

### South America

Most countries affected by COVID-19 in South America imposed quarantine restrictions starting from mid-March to slow down the rapid increase of infections, such as regional lockdowns in Brazil and Chile and national lockdowns in Ecuador, Argentina, and Peru.

**Fig. 17:**
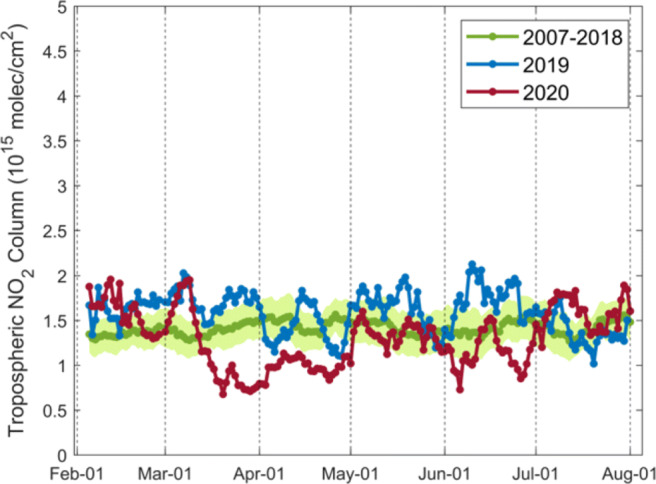
Daily variations in 10-day moving averages of the corrected GOME-2A/B tropospheric NO_2_ columns over Lima in Peru (11^∘^S–13^∘^S, 76^∘^W–78^∘^W) for 2007–2018 (green), 2019 (blue), and 2020 (red). Green shading shows standard error of the mean for 2007–2018. The COVID-19 pandemic lockdown starts on 15 March 2020

Starting on 15 March 2020 with the announcement of one of the earliest and strictest lockdown measures in South America, declines of the GOME-2 tropospheric NO_2_ columns by up to 54.3% are found for the Lima area of Peru in late March and April 2020 in Fig. 17. As a four-step plan on a monthly basis to reopen the economy was announced in early May 2020, the NO_2_ levels return to the normal range and differ within ± 20% afterwards. Compared to TROPOMI data (Fig. S11), the larger noise in the NO_2_ columns from GOME-2 is attributed to the larger effect of the Southern Atlantic Anomaly (SAA), where an anomaly in the Earth’s magnetic field leads to enhanced radiation exposure of the MetOp satellites (Richter et al. 2011; Fioletov et al. 2020).

**Fig. 18:**
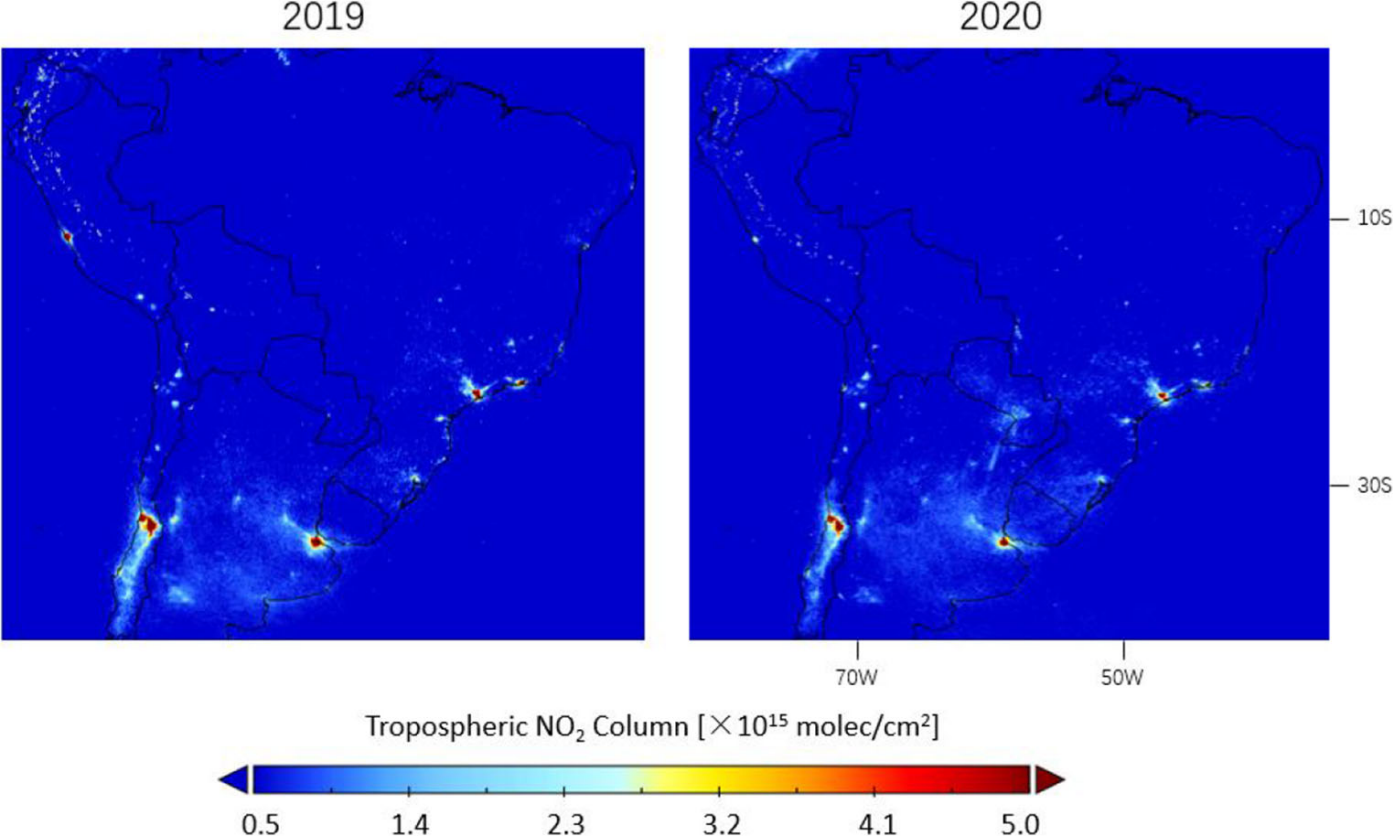
Averages of the corrected tropospheric NO_2_ columns measured by TROPOMI over South America during lockdown in 16 March–15 May in 2020 and comparison with columns in the same time period in 2019

**Fig. 19:**
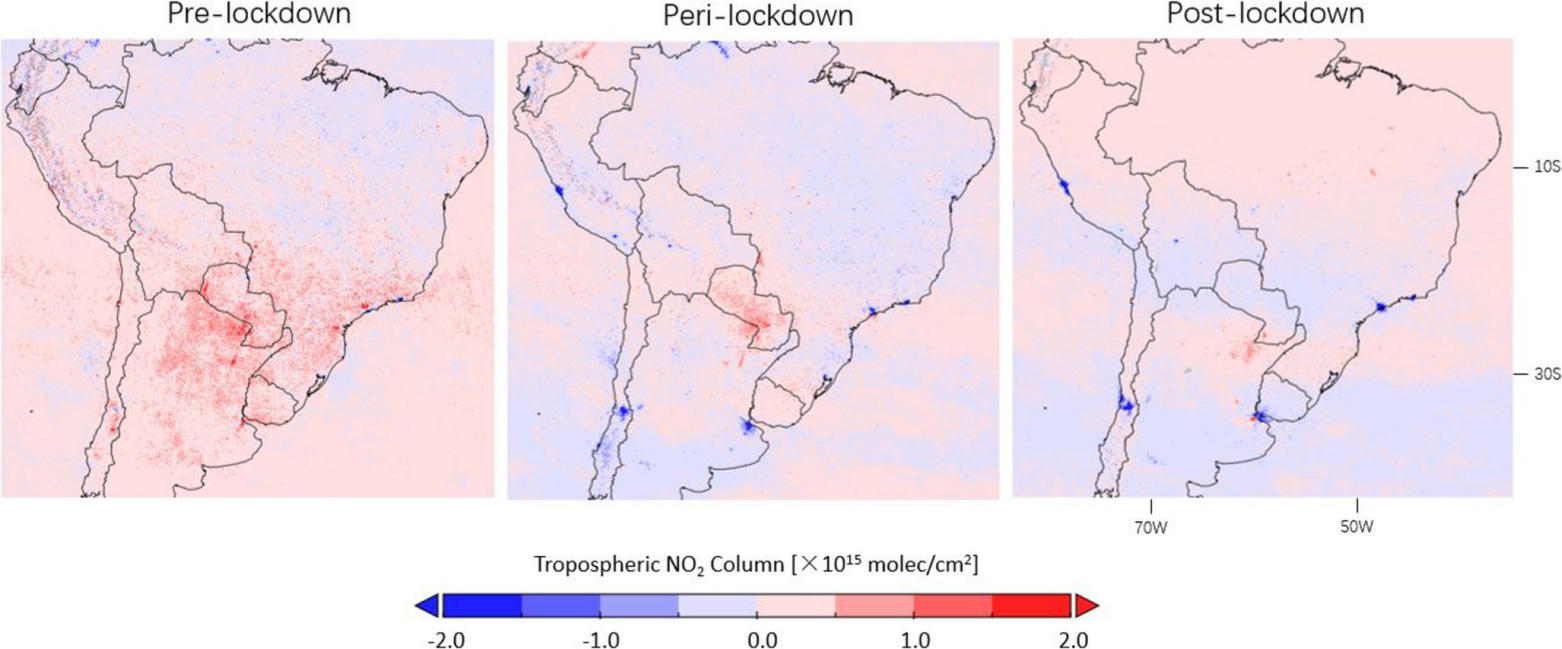
Differences in corrected TROPOMI tropospheric NO_2_ columns between 2020 and 2019 observed before (16 January–15 March), during (16 March–15 May), and after (16 May–15 July) lockdown over South America

The implementation of lockdown measures decreases the TROPOMI tropospheric NO_2_ columns for most South American urban areas in Figs. 18 and 19. Local NO_2_ increases can be attributed to active biomass burning in rural regions, for instance, the NO_2_ enhancements by up to 1 × 10^15^ molec/cm^2^ over Argentina and Paraguay during lockdown are likely related to fires for agricultural use (https://modis.gsfc.nasa.gov/gallery/individual.php?db_date=2020-04-21). Comparing the peri-lockdown NO_2_ drops with pre-lockdown values in Table 1, the declines resulted from the lockdown are 39.1% for Buenos Aires in Argentina, 33.7% for Guayaquil in Ecuador, 81.9% for Lima in Peru, 22.4% for Santiago in Chile, and 59.7% for Sao Paulo in Brazil. During the post-lockdown timeframe, the NO_2_ levels rebound by $\sim $20% in Lima but to lower levels than the pre-lockdown timeframe. A return to the normal NO_2_ level is found for Guayaquil and Buenos Aires, but the comparison in Argentina is complicated due to the increase in fire activity, which is visible from the increased tropospheric NO_2_ columns during the post-lockdown period in Fig. 19 over Paraná River Basin (https://earthobservatory.nasa.gov/images/147031/the-parched-parana-river). While a number of regions remain the current epicenters of the COVID-19 pandemic, regional studies with longer NO_2_ time series will be the subject of future work.

## Conclusion

In response to the rapid COVID-19 spread, countries around the world have imposed lockdown restrictions. Quantifying the temporal changes of pollutant concentrations due to COVID-19 restrictions is important to understand the impact of public health measures on environment, economy, and society.

Mainly emitted anthropogenically from the road transport and industrial activities, the variations of tropospheric NO_2_ columns are analyzed based on the long-term global dataset (since 2007) from GOME-2 with a morning overpass and the high-resolution measurements (5.5 km×3.5 km) from TROPOMI with an early afternoon overpass. The GOME-2 and TROPOMI NO_2_ data are retrieved in a harmonized manner and corrected for trend, season, and meteorology using a statistical method.

With good consistency between GOME-2 and TROPOMI measurements, strong decreases in tropospheric NO_2_ columns are observed during the lockdown period not only in comparison with levels before and after the lockdown but also compared to identical periods in the historical data. China observes an average 24.1% decline of NO_2_ levels due to the pollution control policies and a further reduction of $\sim $30% due to the COVID-19 containment measures after the Chinese New Year holiday in late January 2020. The NO_2_ amount gradually returns to the normal level as previous years after 2 months of lockdown. Similar decline and rebound are observed for southern European countries such as Italy, Portugal, and Spain, where the mean NO_2_ decline because of emission control is $\sim $20% and the lockdown-related drop during mid-March to mid-May is $\sim $30%. In India, the tropospheric NO_2_ columns decrease by 42% on average for populated areas and by up to 14.2% for particular power plant locations, followed by a rebound in late June after 3 months of lockdown. The USA reports a lockdown-related NO_2_ reduction of 34.9% on average for western regions such as California and up to 17.8% for eastern areas. In South America, the tropospheric NO_2_ columns reduce by up to 81.9% during mid-March to mid-May due to the lockdown.

In conclusion, the NO_2_ drops due to the lockdown restrictions are estimated to be 30% for populated cities in China and southern Europe, 42% in India, 35% in the southwestern USA, and 48% in South America. Due to the recovery of social and economic activities in a phased manner, gradual rebounds of the tropospheric NO_2_ columns to normal levels are found for countries such as China, Italy, and India. As the lockdown is still ongoing for a number of regions worldwide in response to the second wave of outbreak, and its long-term effect on NO_2_ variations (e.g., due to the possible economic downturn) is uncertain, a further monitoring of the NO_2_ concentration recovery will be necessary.

## Electronic supplementary material

Below is the link to the electronic supplementary material.

(DOCX 7.75 MB)
