# Supplementary material for: Nitrogen dioxide decline and rebound observed by GOME-2 and TROPOMI during COVID-19 pandemic
Source: Air Qual Atmos Health. 2021 Aug 28;14(11):1737–55. doi: 10.1007/s11869-021-01046-2 (PMC8397874; doi:10.1007/s11869-021-01046-2)
Supplement: Supplementary file 1 — (DOCX 7.75 MB) [file 11869_2021_1046_MOESM1_ESM.docx]

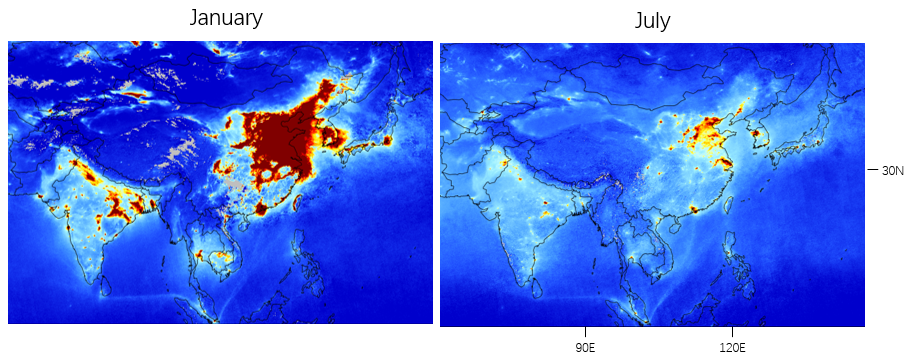

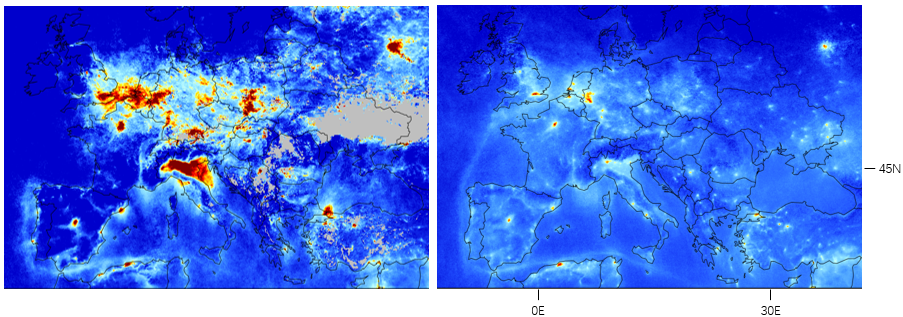

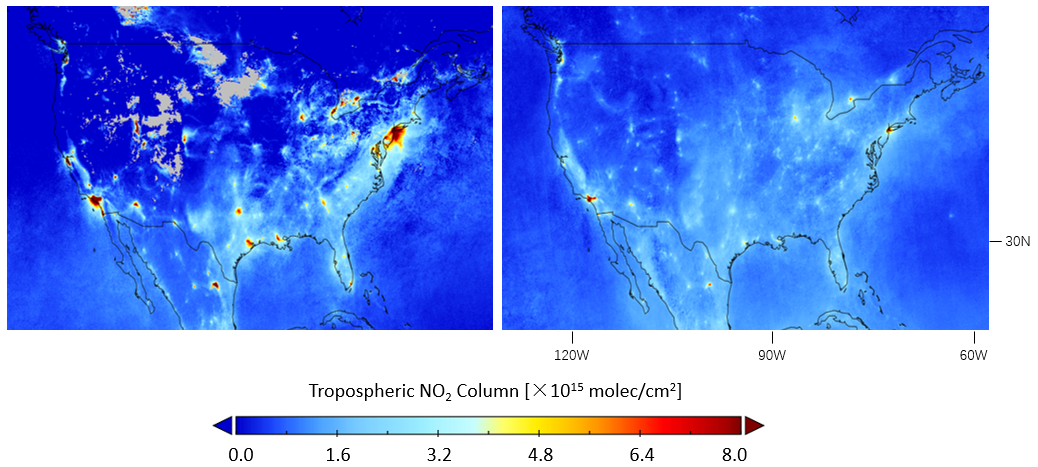


Figure S1. Monthly mean tropospheric NO_2_ columns measured by TROPOMI over Asia, Europe, and North America in January and July 2019. Results are retrieved using a harmonized algorithm from DLR as introduced in Sect. 2.3.


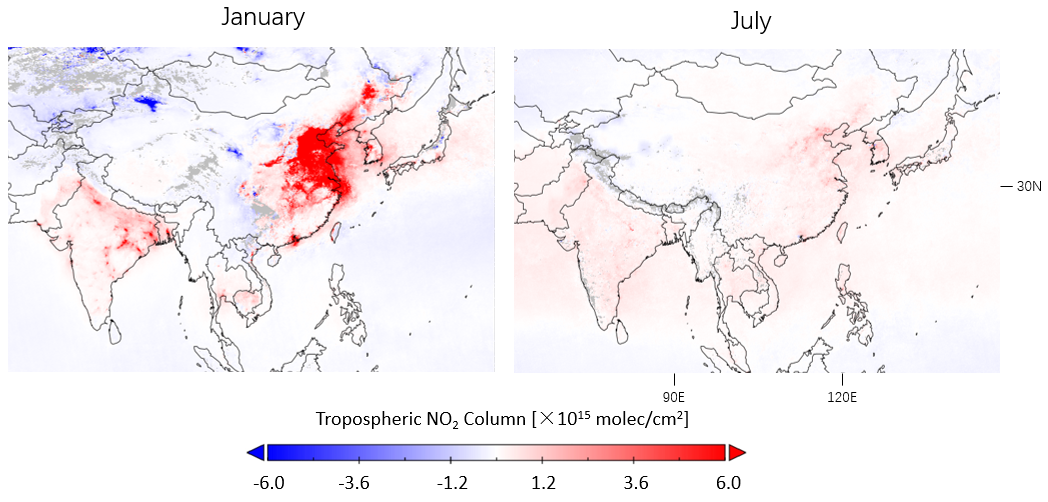

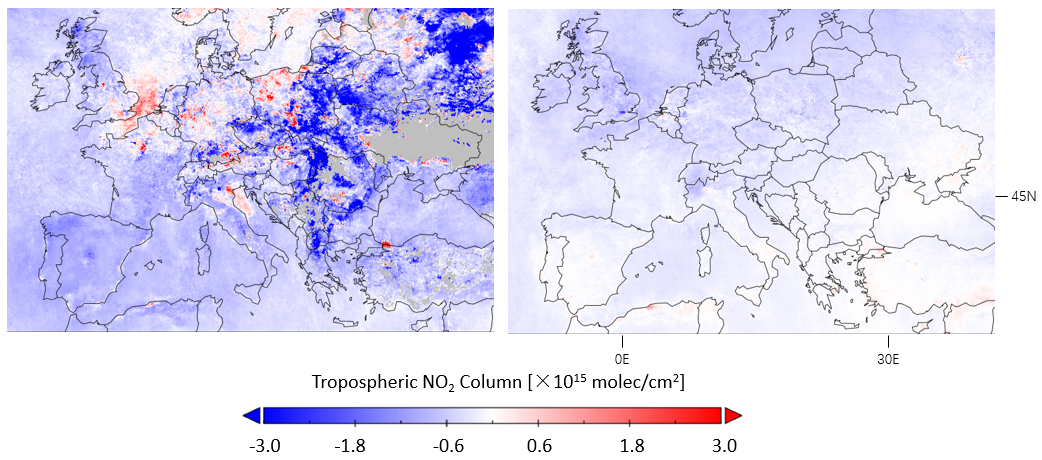


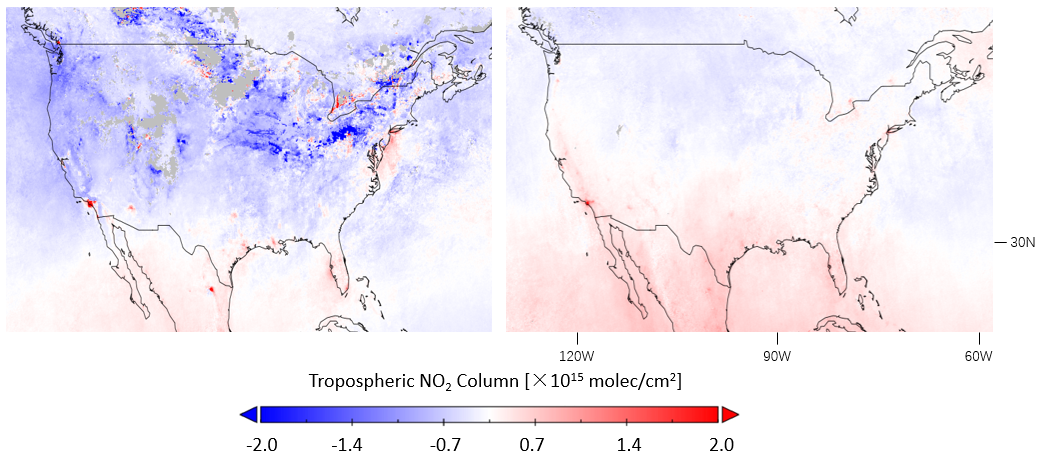


Figure S2. Differences in TROPOMI monthly mean tropospheric NO_2_ columns retrieved using a harmonized algorithm from DLR and the operational implementation from KNMI (DLR-KNMI) over Asia, Europe, and North America in January and July 2019.


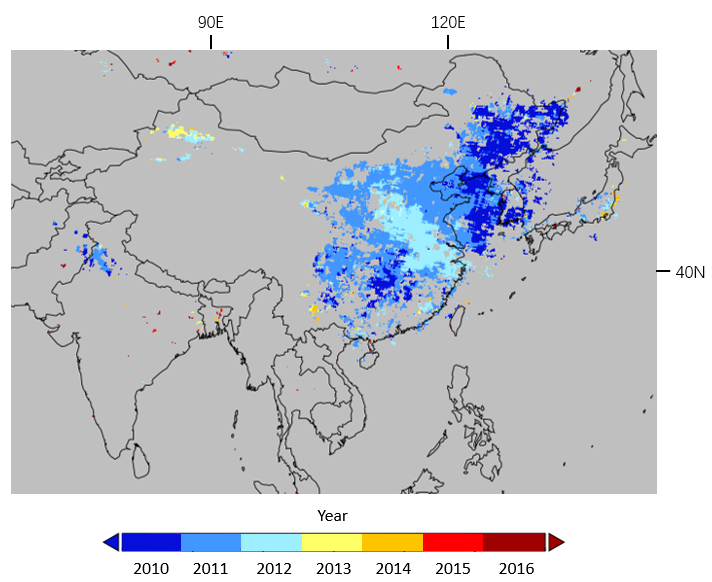


Figure S3. Year of tropospheric NO_2_ column trend reversal from positive to negative over Asia. Only grid cells with a statistically significant trend at the 95% confidence level for the period before or after the year of reversal and with a long-term tropospheric NO_2_ column average larger than 1×10^15^ molecules/cm^2^ are shown.

India


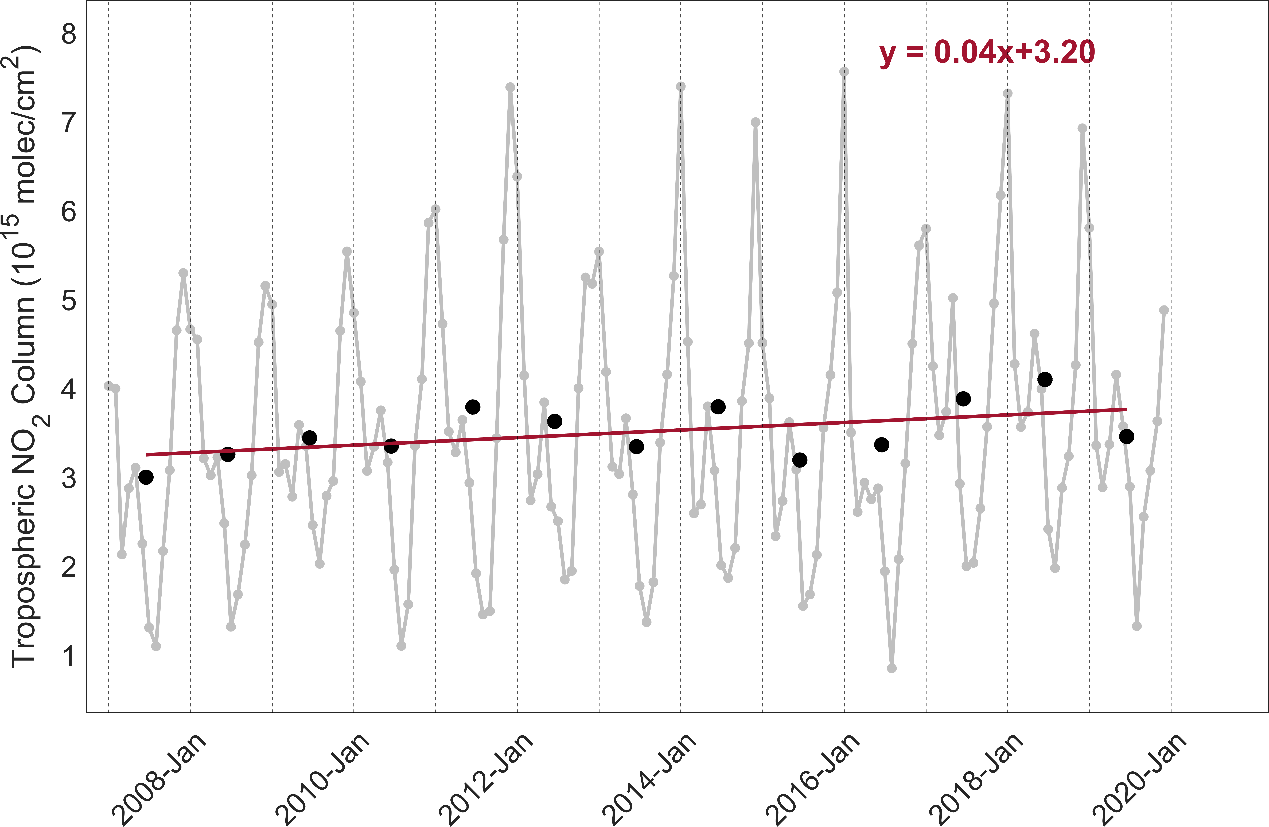


U.S.


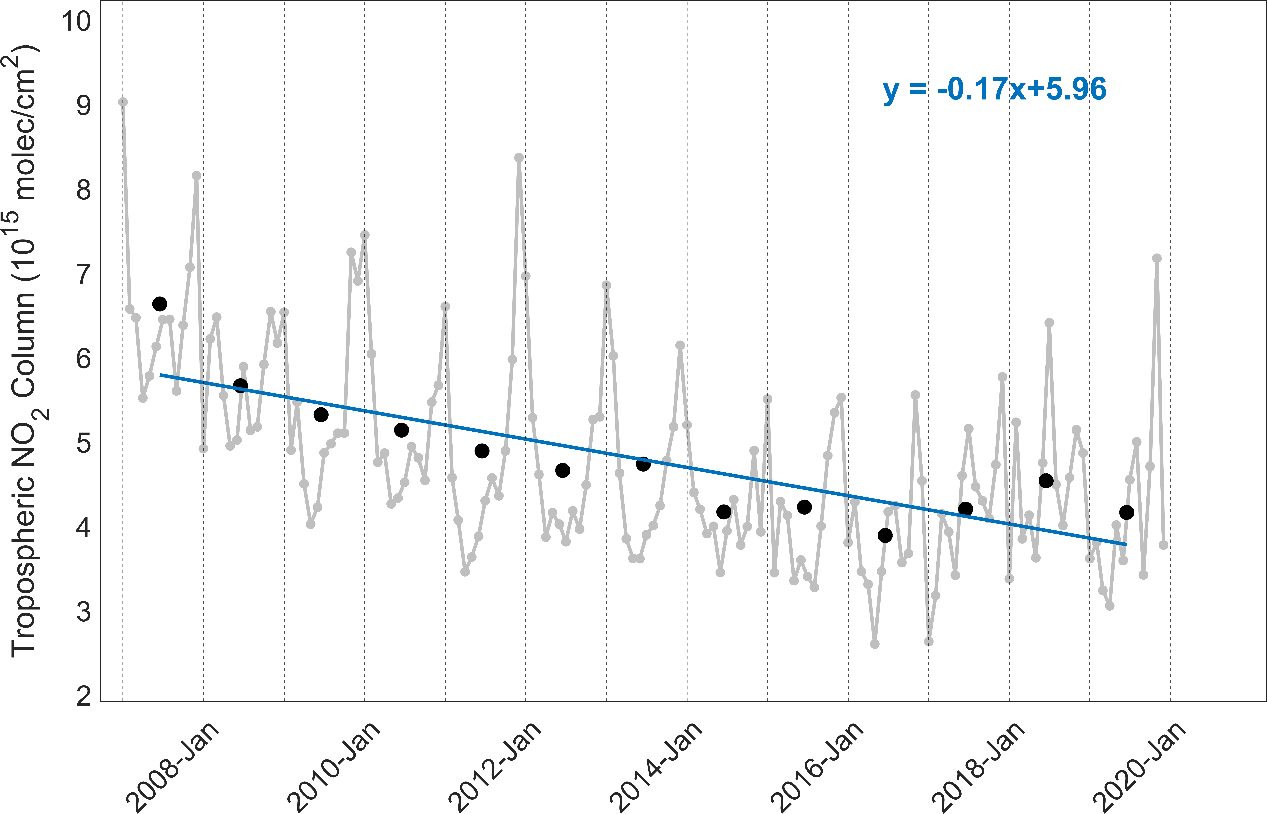


Figure S4. Time series of monthly (gray line) and yearly (black dots) mean tropospheric NO_2_ columns measured by GOME-2A over New Delhi in northern India (27.6°N-29.6°N, 76.2°E-78.2°E), Los Angeles in the southwestern U.S. (33.5°N-35.5°N, 117.25°W-119.25°W), and Lima in Peru (11°S-13°S, 76°W-78°W). The linear fitting results for the yearly averages are also shown. (Figure continues on next page.)

Peru


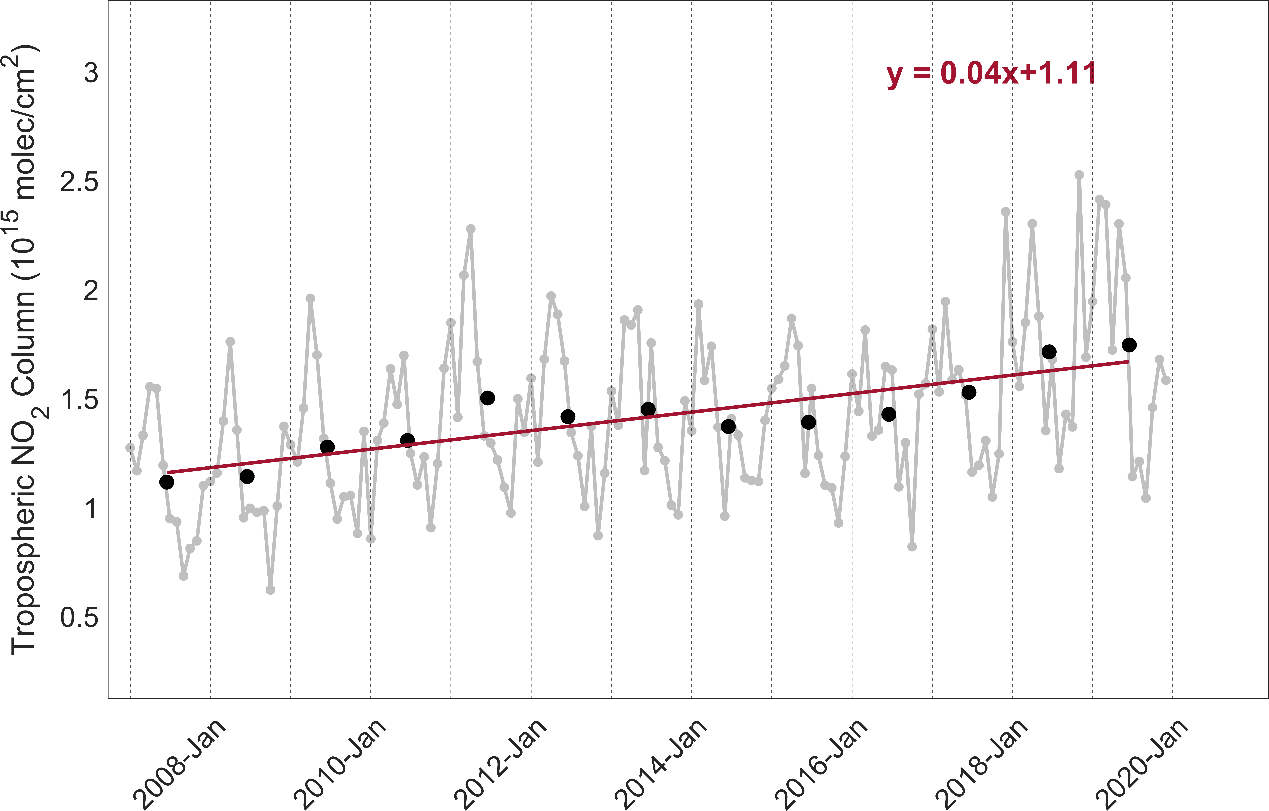


Figure S4. (continued)


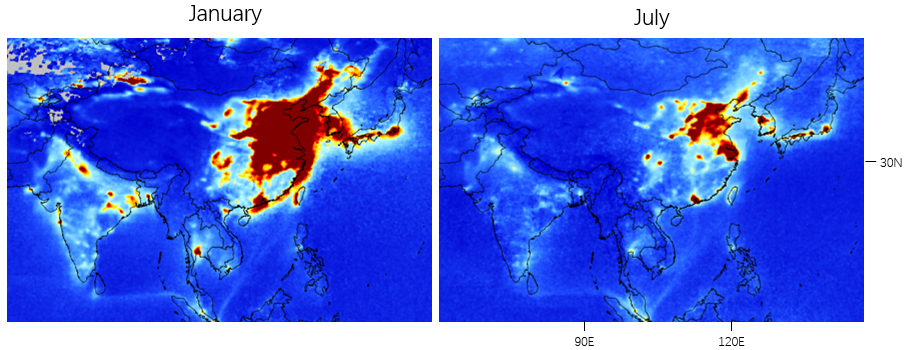

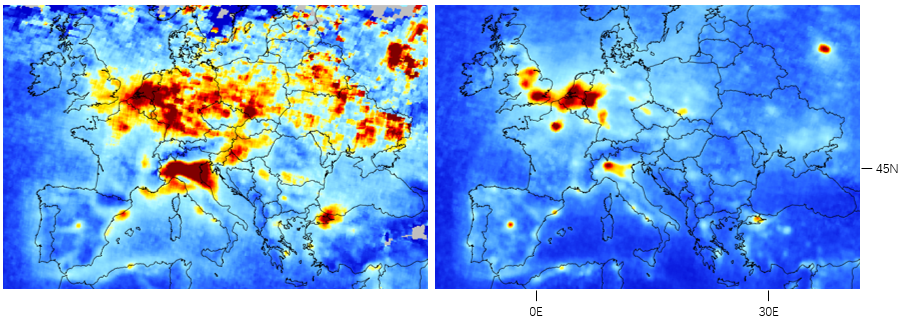

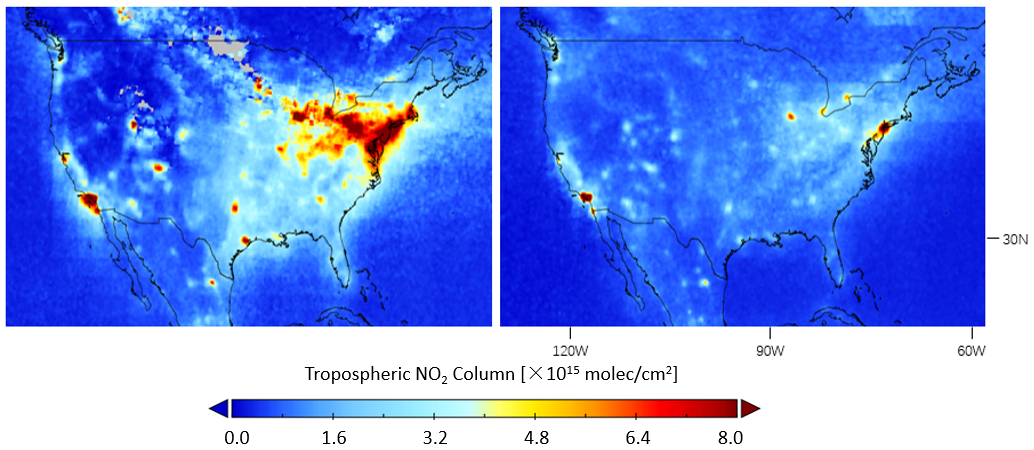


Figure S5. 13-year (2007-2019) monthly mean tropospheric NO_2_ columns measured by GOME-2A over Asia, Europe, and North America in January and July.


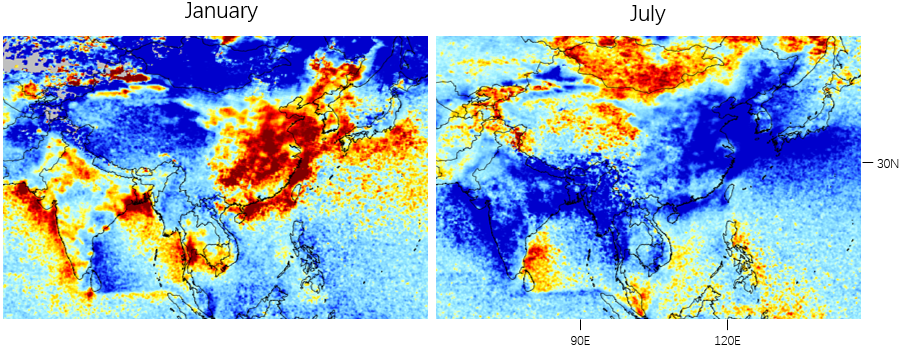

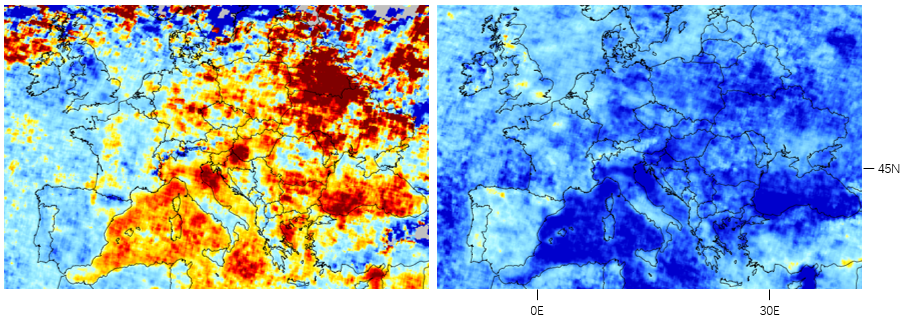

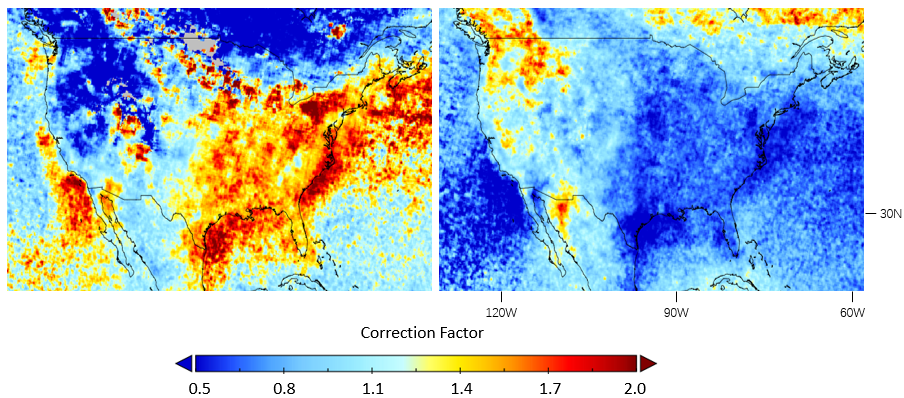


Figure S6. GOME-2A season correction factors over Asia, Europe, and North America in January and July.


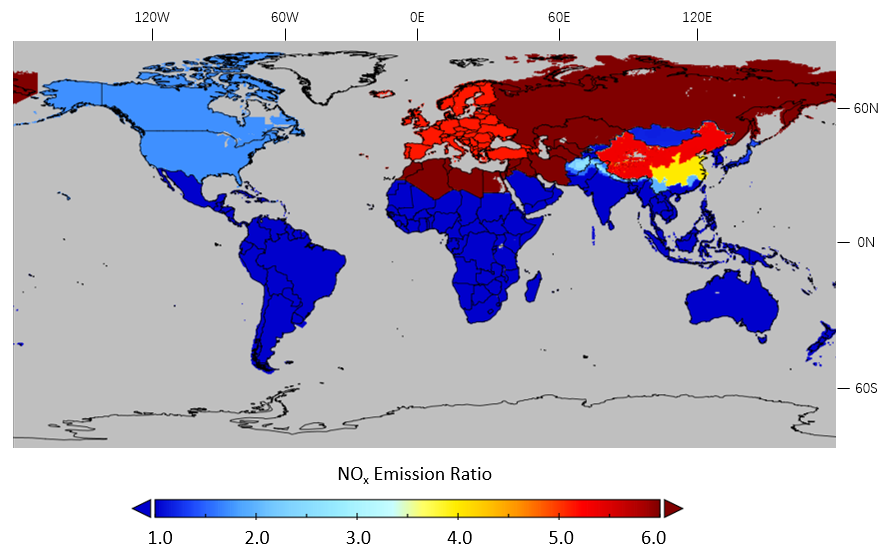


Figure S7. EDGAR-HTAP_V2 monthly mean NO_x_ emissions contributed by the residential sector (<https://edgar.jrc.ec.europa.eu/htap_v2/>) in January 2010 compared with their levels in July 2010 (ratio between months).

China


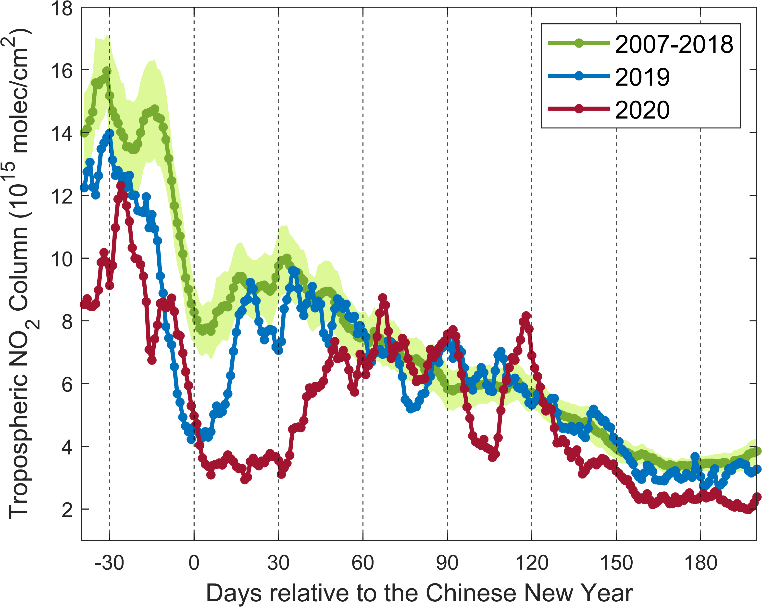


Italy


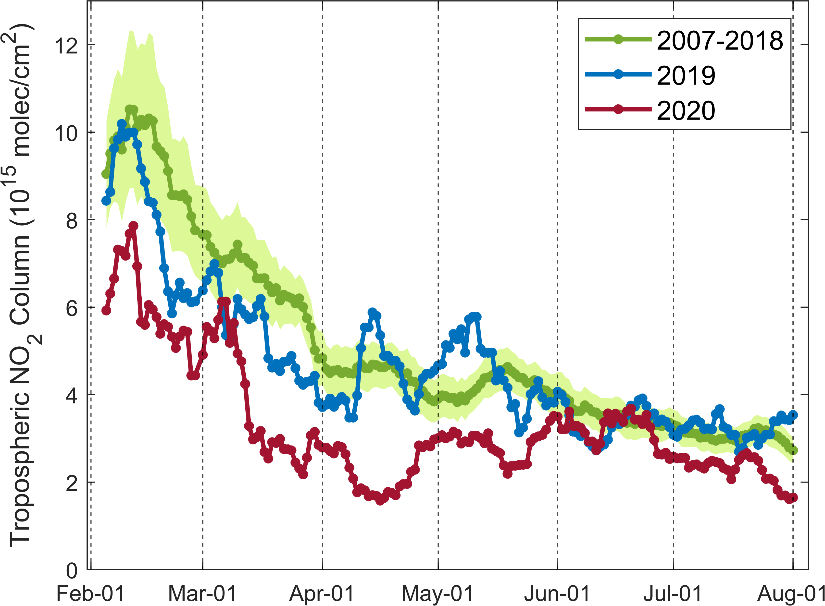


India


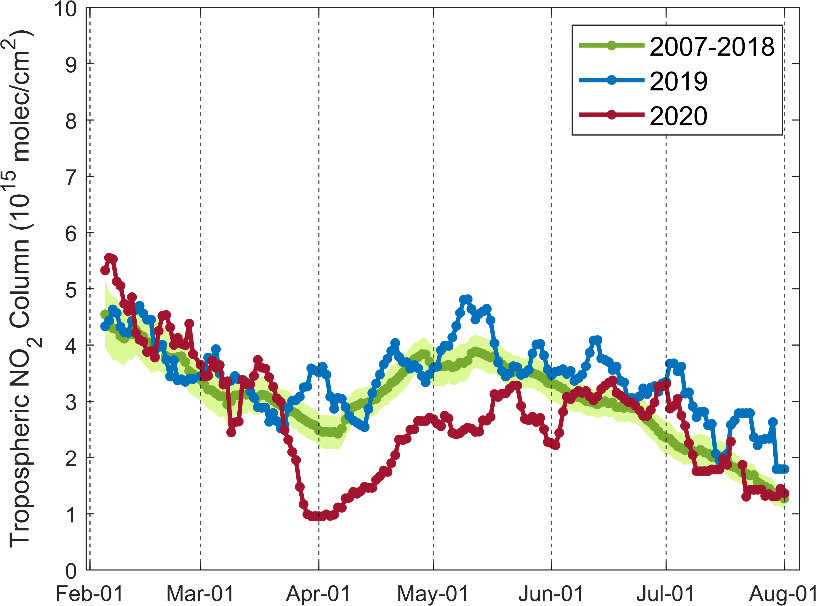


U.S.


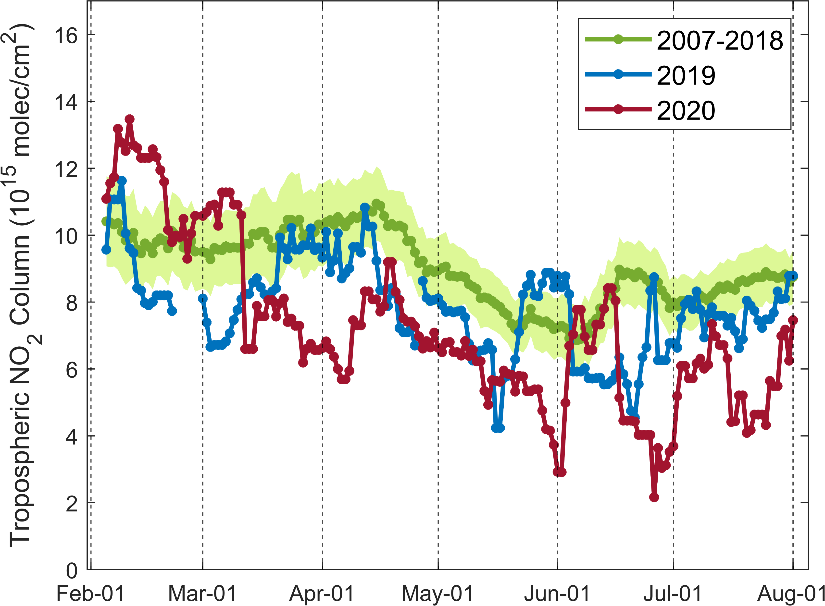


Peru


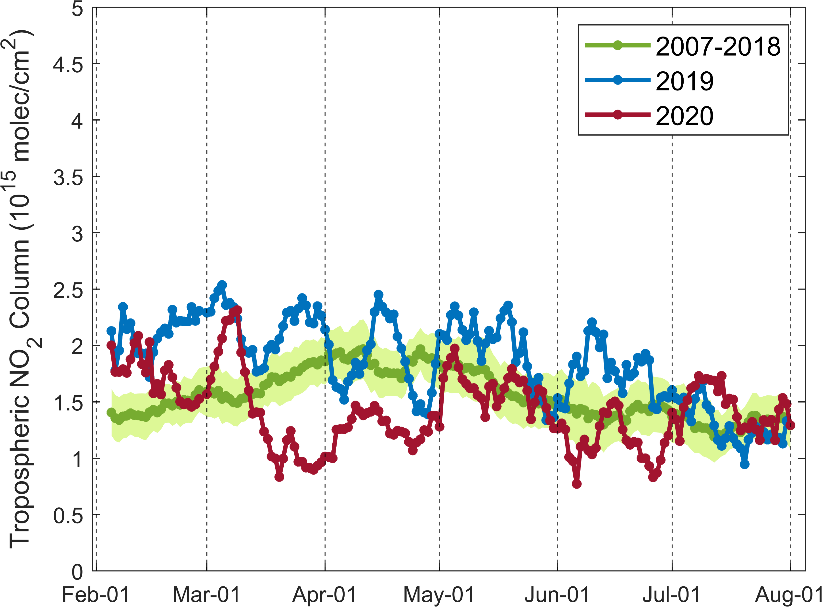


Figure S8. Daily variations in 10-day moving averages of the original uncorrected GOME-2A/B tropospheric NO_2_ columns in 2007-2018 (green), 2019 (blue), and 2020 (red). Results are shown for eastern China (21°N-41°N, 110°E-122°E), northern Italy (45°N-46.5°N, 7°E-13°E), New Delhi in northern India (27.6°N-29.6°N, 76.2°E-78.2°E), Los Angeles in the southwestern U.S. (33.5°N-35.5°N, 117.25°W-119.25°W), and Lima in Peru (11°S-13°S, 76°W-78°W). Green shading shows standard error of the mean for 2007-2018. The annually varying dates of the Chinese New Year is accounted for.

China


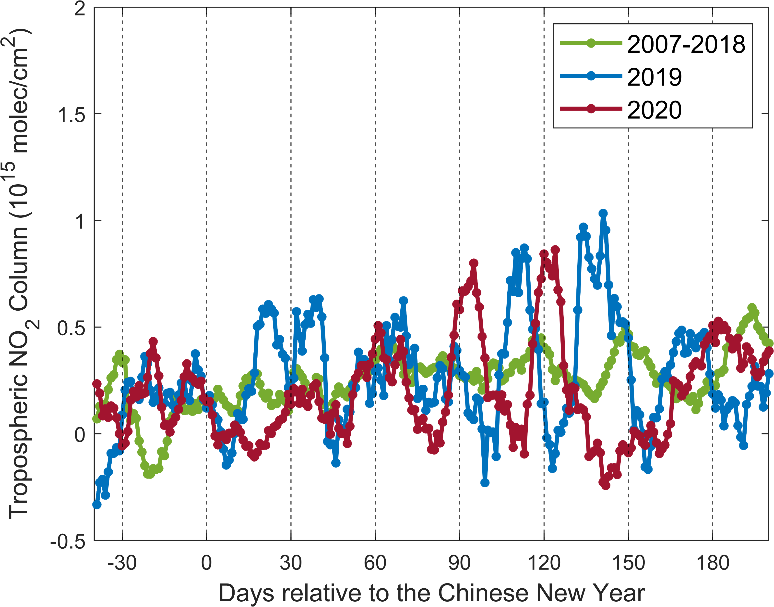


Italy


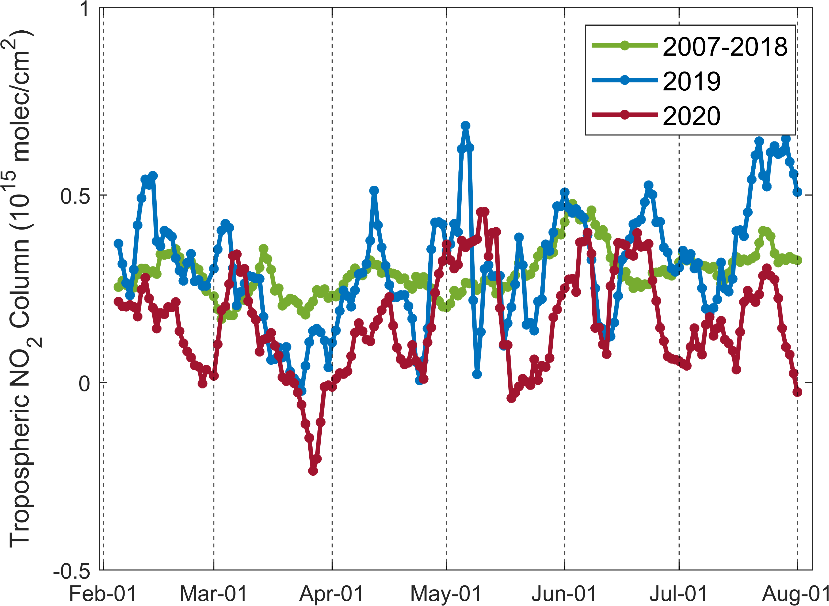


India


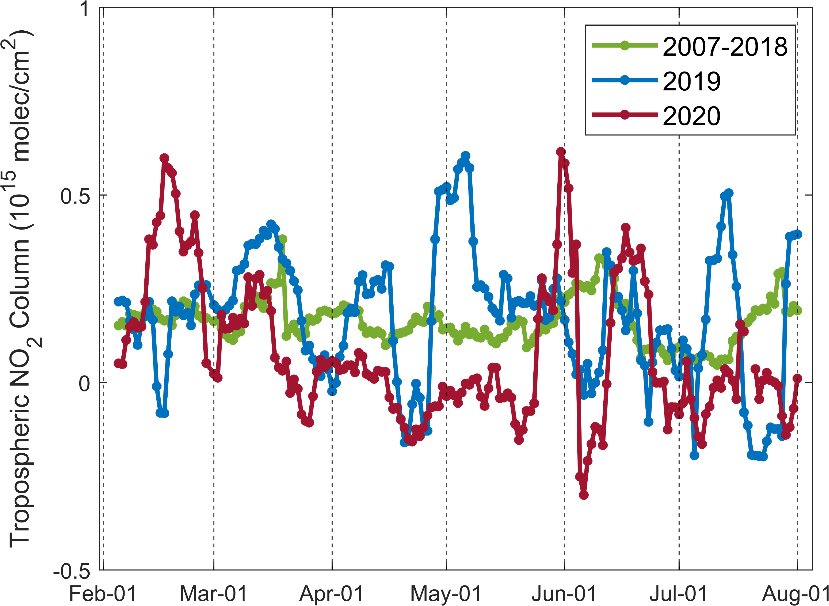


U.S.


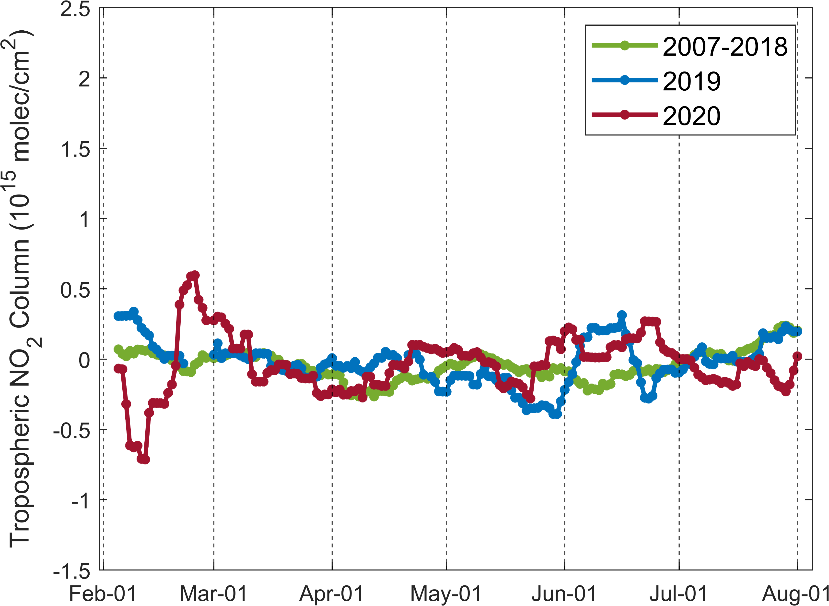


Peru


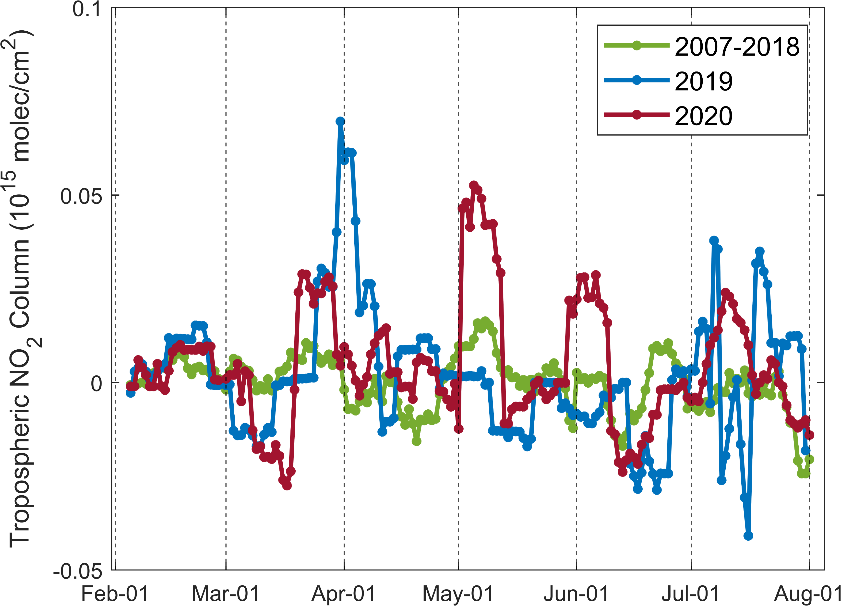


Figure S9. Differences in GOME-2A/B tropospheric NO_2_ column 10-day moving averages with and without (with-without) meteorological corrections in 2007-2018 (green), 2019 (blue), and 2020 (red). The dependencies on trend and season are considered. Results are shown for eastern China (21°N-41°N, 110°E-122°E), northern Italy (45°N-46.5°N, 7°E-13°E), New Delhi in northern India (27.6°N-29.6°N, 76.2°E-78.2°E), Los Angeles in the southwestern U.S. (33.5°N-35.5°N, 117.25°W-119.25°W), and Lima in Peru (11°S-13°S, 76°W-78°W). The annually varying dates of the Chinese New Year is accounted for.

China


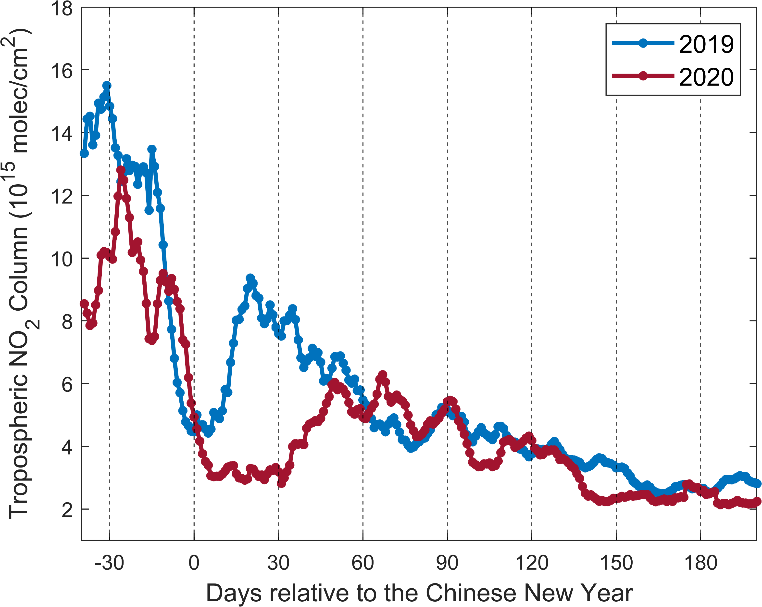


Italy


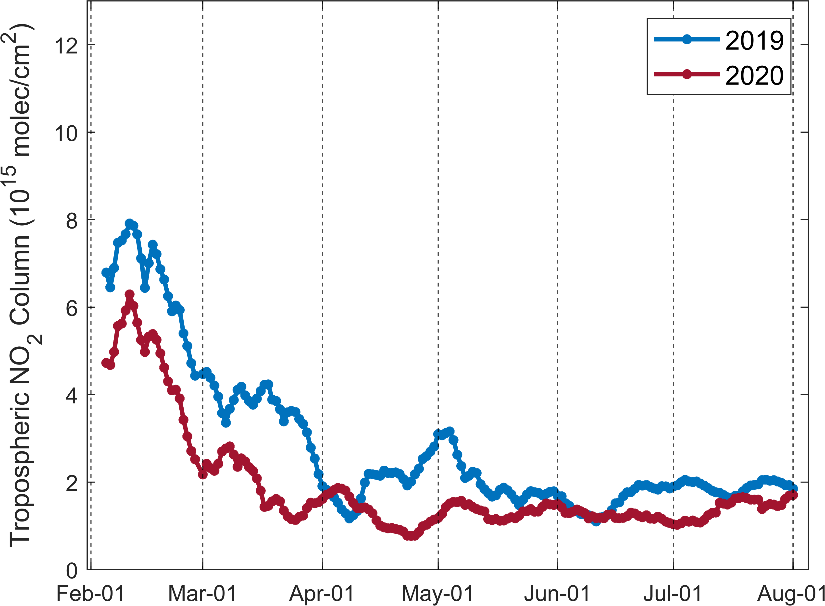


India


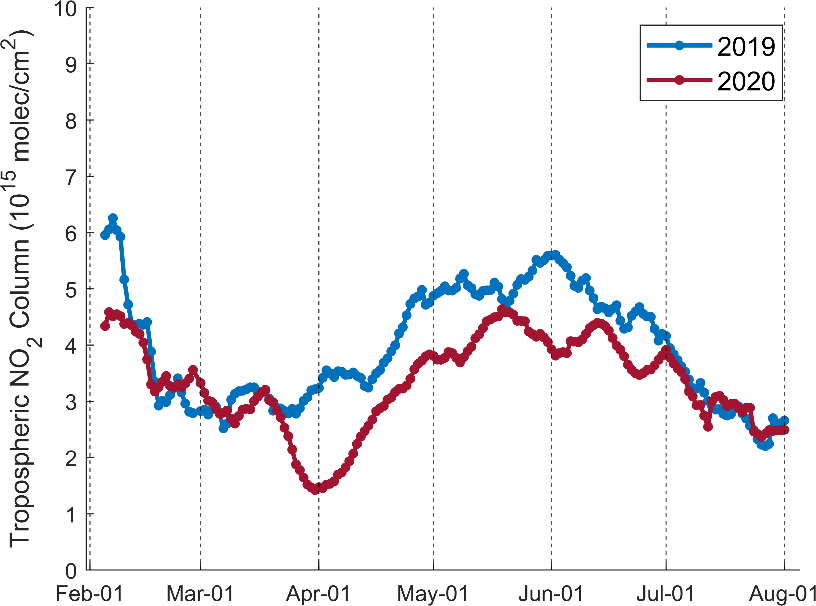


U.S.


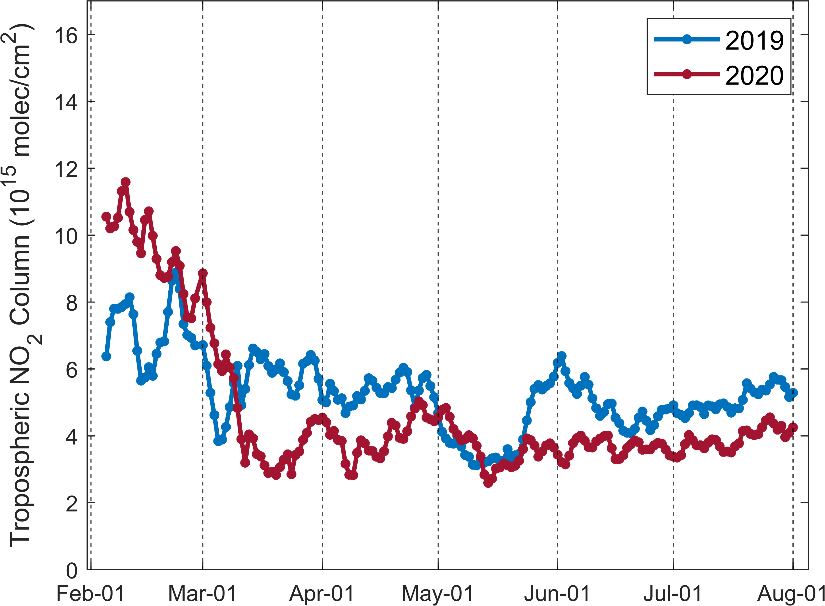


Peru


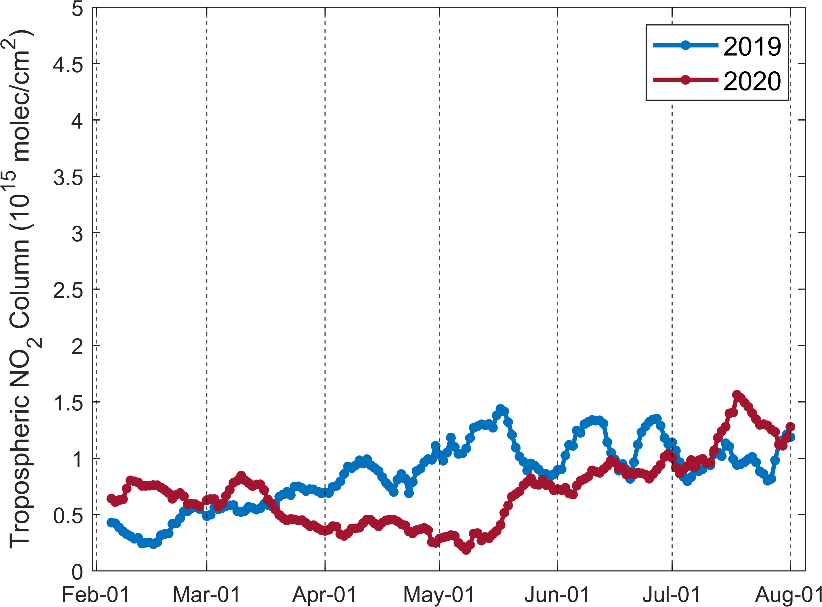


Figure S10. Daily variations in 10-day moving averages of the original uncorrected TROPOMI tropospheric NO_2_ columns in 2019 (blue) and 2020 (red). Results are shown for eastern China (21°N-41°N, 110°E-122°E), northern Italy (45°N-46.5°N, 7°E-13°E), New Delhi in northern India (27.6°N-29.6°N, 76.2°E-78.2°E), Los Angeles in the southwestern U.S. (33.5°N-35.5°N, 117.25°W-119.25°W), and Lima in Peru (11°S-13°S, 76°W-78°W). The annually varying dates of the Chinese New Year is accounted for.

China


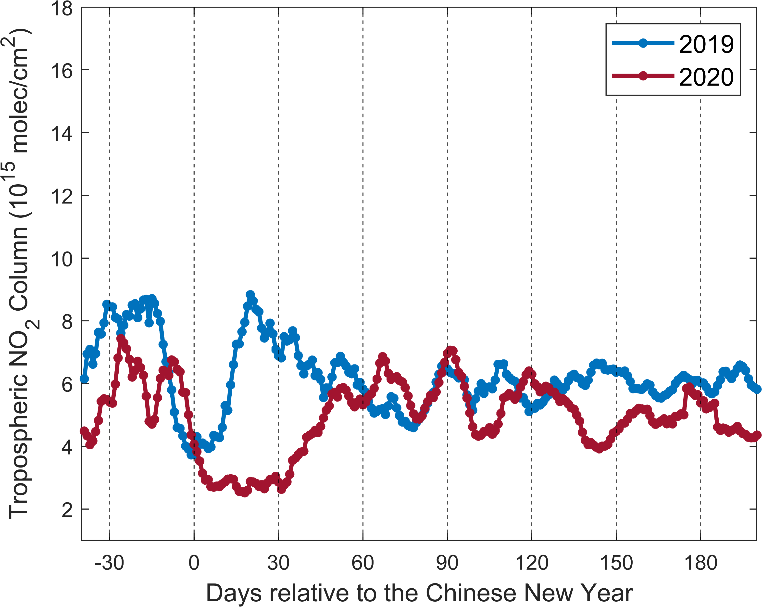


Italy


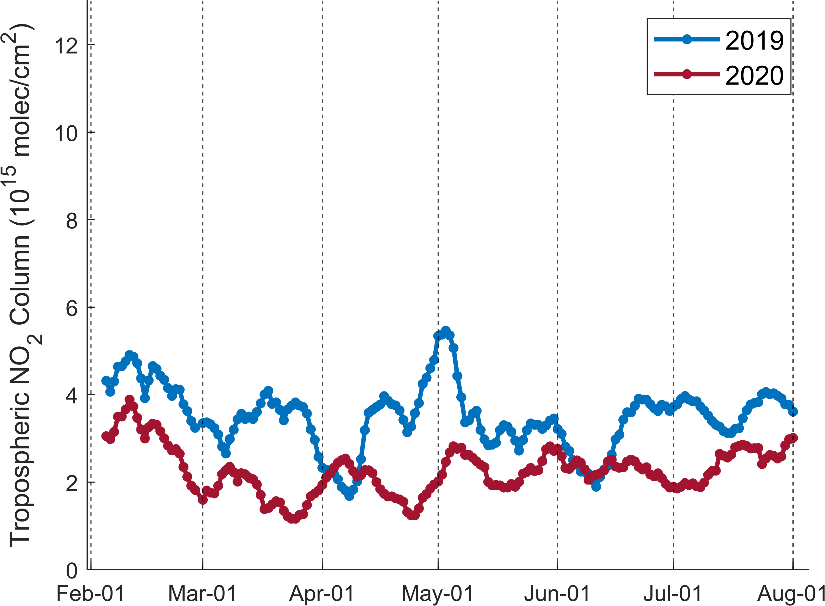


India


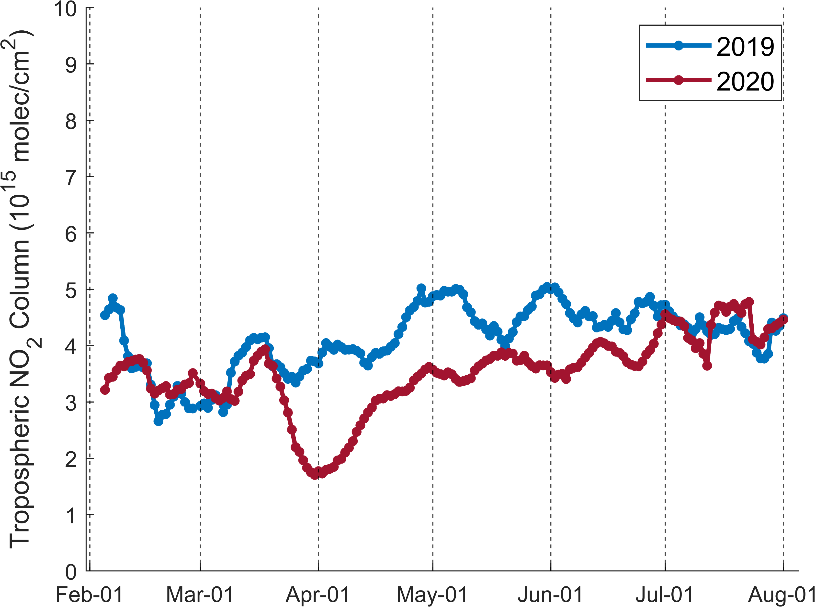


U.S.


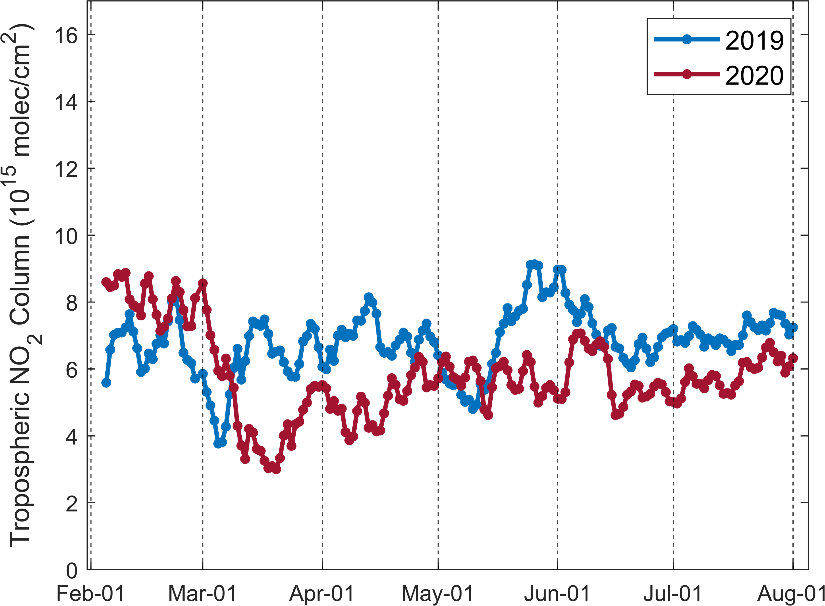


Peru


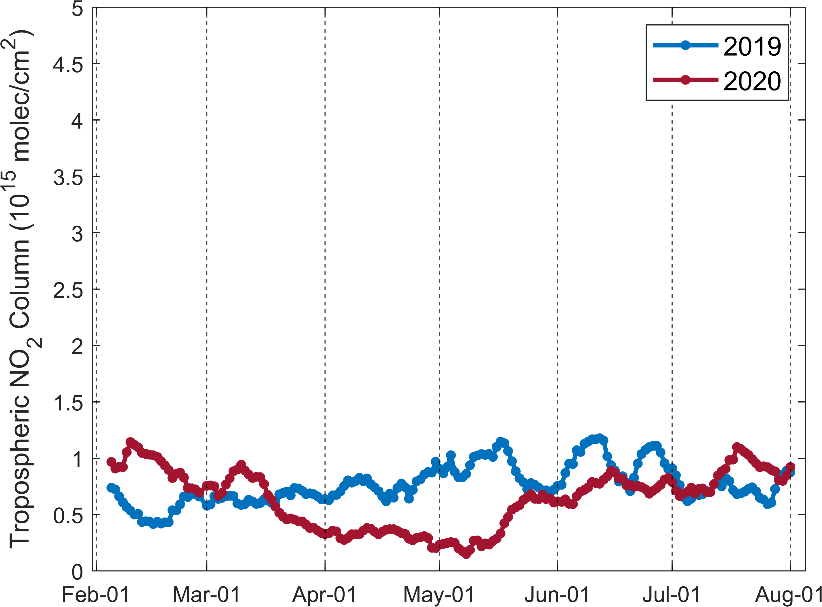


Figure S11. Similar to Fig. S10 but for TROPOMI tropospheric NO_2_ columns with corrections for trend, season, and meteorology.

China


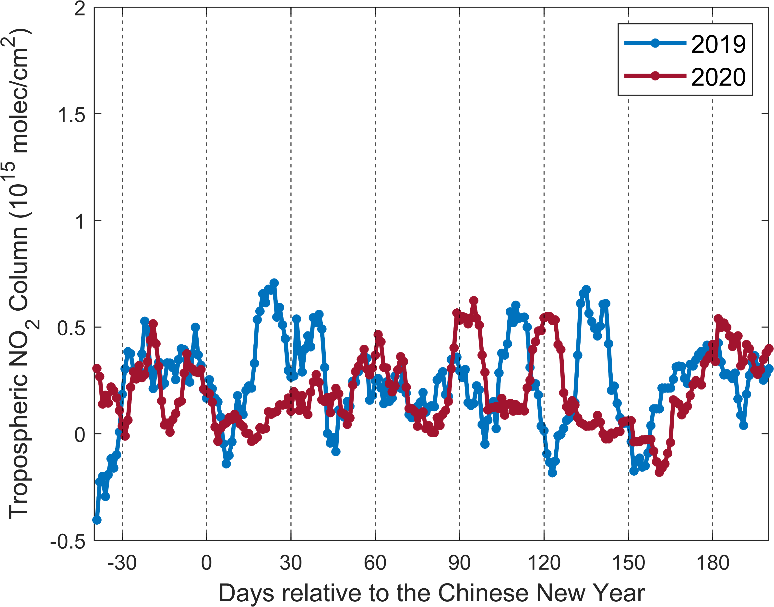


Italy


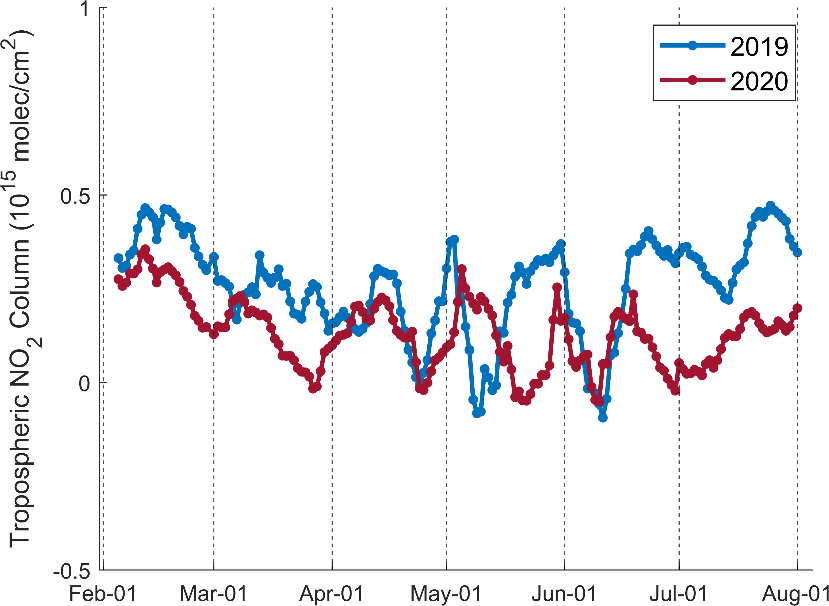


India


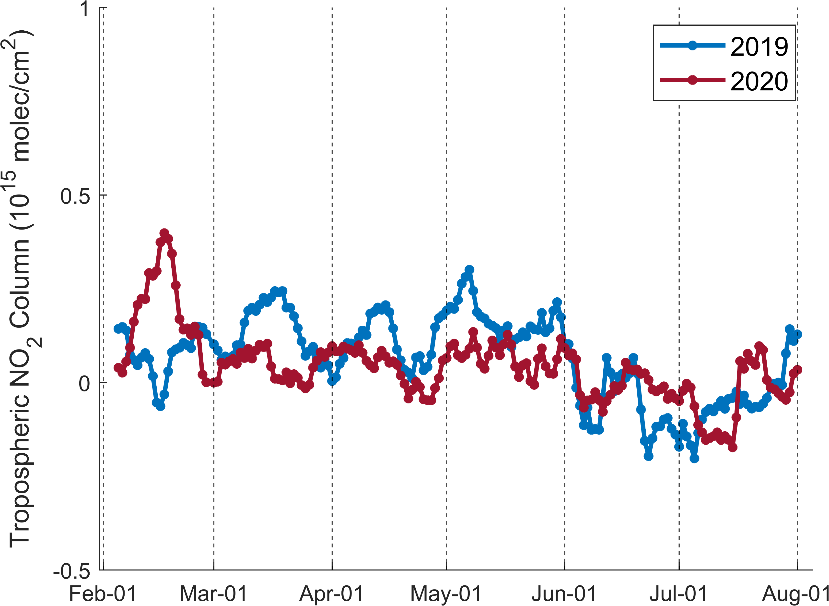


U.S.


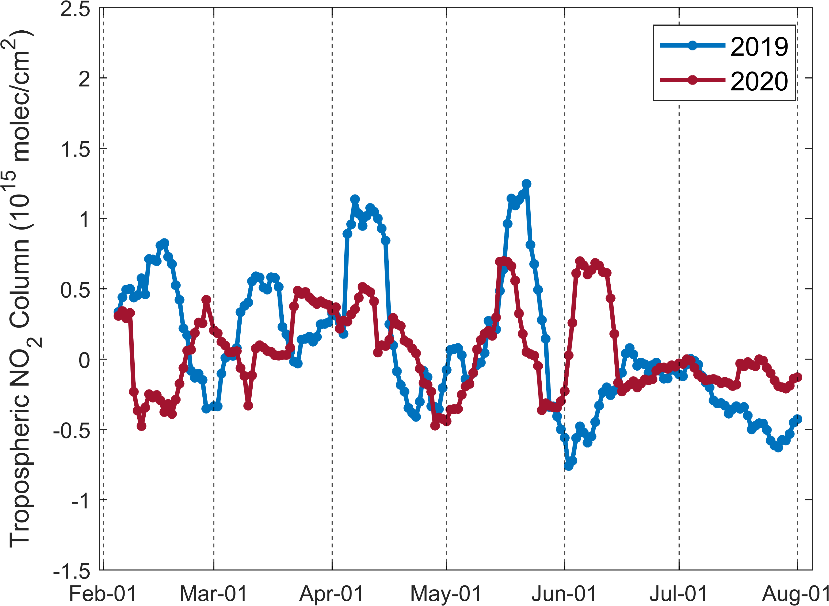


Peru


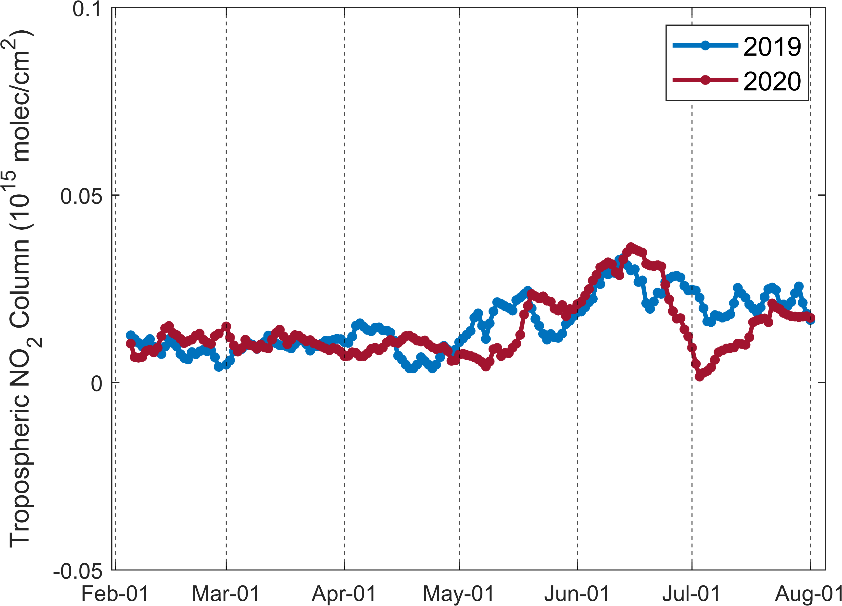


Figure S12. Differences in TROPOMI tropospheric NO_2_ column 10-day moving averages with and without (with-without) meteorological corrections in 2019 (blue) and 2020 (red). The dependencies on trend and season are considered. Results are shown for eastern China (21°N-41°N, 110°E-122°E), northern Italy (45°N-46.5°N, 7°E-13°E), New Delhi in northern India (27.6°N-29.6°N, 76.2°E-78.2°E), Los Angeles in the southwestern U.S. (33.5°N-35.5°N, 117.25°W-119.25°W), and Lima in Peru (11°S-13°S, 76°W-78°W). The annually varying dates of the Chinese New Year is accounted for.
